# Sensory salience processing moderates attenuated gazes on faces in autism spectrum disorder: a case–control study

**DOI:** 10.1186/s13229-023-00537-6

**Published:** 2023-02-09

**Authors:** Nico Bast, Luke Mason, Christine Ecker, Sarah Baumeister, Tobias Banaschewski, Emily J. H. Jones, Declan G. M. Murphy, Jan K. Buitelaar, Eva Loth, Gahan Pandina, the EU-AIMS LEAP Group, Jumana Ahmad, Sara Ambrosino, Bonnie Auyeung, Tobias Banaschewski, Simon Baron-Cohen, Nico Bast, Sarah Baumeister, Christian F. Beckmann, Sven Bölte, Thomas Bourgeron, Carsten Bours, Michael Brammer, Daniel Brandeis, Claudia Brogna, Yvette de Bruijn, Jan K. Buitelaar, Bhismadev Chakrabarti, Tony Charman, Ineke Cornelissen, Daisy Crawley, Flavio Dell’Acqua, Guillaume Dumas, Sarah Durston, Christine Ecker, Jessica Faulkner, Vincent Frouin, Pilar Garcés, David Goyard, Lindsay Ham, Hannah Hayward, Joerg Hipp, Rosemary Holt, Mark Johnson, Emily J. H. Jones, Prantik Kundu, Meng-Chuan Lai, Xavier Liogier D’ardhuy, Michael V. Lombardo, Eva Loth, David J. Lythgoe, René Mandl, Andre Marquand, Luke Mason, Maarten Mennes, Andreas Meyer-Lindenberg, Carolin Moessnang, Declan G. M. Murphy, Bethany Oakley, Laurence O’Dwyer, Marianne Oldehinkel, Bob Oranje, Gahan Pandina, Antonio M. Persico, Barbara Ruggeri, Amber Ruigrok, Jessica Sabet, Roberto Sacco, Antonia San José Cáceres, Emily Simonoff, Will Spooren, Julian Tillmann, Roberto Toro, Heike Tost, Jack Waldman, Steve C. R. Williams, Caroline Wooldridge, Marcel P. Zwiers, Christine M. Freitag

**Affiliations:** 1 Department of Child and Adolescent Psychiatry, Psychosomatics and Psychotherapy, Autism Research and Intervention Center of Excellence, University Hospital Frankfurt, Goethe-University, Deutschordenstraße 50, 60528 Frankfurt Am Main, Germany; 2 Centre for Brain and Cognitive Development, Birkbeck College, University of London, Malet Street, London, UK; 3 Department of Child and Adolescent Psychiatry, Central Institute of Mental Health, Medical Faculty Mannheim, University of Heidelberg, Mannheim, Germany; 4 Institute of Psychiatry, Psychology and Neuroscience, King’s College, London, London, UK; 5 Department of Cognitive Neuroscience, Donders Institute for Brain, Cognition and Behaviour, Radboud University Medical Center, Nijmegen, The Netherlands; 6 Janssen Research & Development, 1125 Trenton Harbourton Road, Titusville, NJ 08560 USA

**Keywords:** Locus coeruleus, ASD, Pupillometry, Saliency maps, Computer vision, Eye tracking, Norepinephrine, Social attention, Visual exploration, Naturalistic visual attention

## Abstract

**Background:**

Attenuated social attention is a key marker of autism spectrum disorder (ASD). Recent neuroimaging findings also emphasize an altered processing of sensory salience in ASD. The locus coeruleus–norepinephrine system (LC-NE) has been established as a modulator of this sensory salience processing (SSP). We tested the hypothesis that altered LC-NE functioning contributes to different SSP and results in diverging social attention in ASD.

**Methods:**

We analyzed the baseline eye-tracking data of the EU-AIMS Longitudinal European Autism Project (LEAP) for subgroups of autistic participants (*n* = 166, age = 6–30 years, IQ = 61–138, gender [female/male] = 41/125) or neurotypical development (TD; *n* = 166, age = 6–30 years, IQ = 63–138, gender [female/male] = 49/117) that were matched for demographic variables and data quality. Participants watched brief movie scenes (*k* = 85) depicting humans in social situations (human) or without humans (non-human). SSP was estimated by gazes on physical and motion salience and a corresponding pupillary response that indexes phasic activity of the LC-NE. Social attention is estimated by gazes on faces via manual areas of interest definition. SSP is compared between groups and related to social attention by linear mixed models that consider temporal dynamics within scenes. Models are controlled for comorbid psychopathology, gaze behavior, and luminance.

**Results:**

We found no group differences in gazes on salience, whereas pupillary responses were associated with altered gazes on physical and motion salience. In ASD compared to TD, we observed pupillary responses that were higher for non-human scenes and lower for human scenes. In ASD, we observed lower gazes on faces across the duration of the scenes. Crucially, this different social attention was influenced by gazes on physical salience and moderated by pupillary responses.

**Limitations:**

The naturalistic study design precluded experimental manipulations and stimulus control, while effect sizes were small to moderate. Covariate effects of age and IQ indicate that the findings differ between age and developmental subgroups.

**Conclusions:**

Pupillary responses as a proxy of LC-NE phasic activity during visual attention are suggested to modulate sensory salience processing and contribute to attenuated social attention in ASD.

**Supplementary Information:**

The online version contains supplementary material available at 10.1186/s13229-023-00537-6.

## Background

Visual attention is driven by relevant stimuli [1] that are defined by an interaction of sensory salience and semantic content [2]. Sensory salience describes conspicuous stimulus features [3], whereas semantic content describes acquired knowledge about stimuli [4]. Human faces are particularly relevant and drive human visual attention [5]. Autism spectrum disorder (ASD) is characterized by an attenuated visual attention to human faces [6]. This has usually been explained by an altered semantic content of social stimuli such as an attenuated reward attribution [7, 8]. However, different processing of sensory salience in ASD might also drive attenuated visual attention to human faces. This opposing hypothesis is tested in the current study.

We define this sensory salience processing (SSP) as the consideration of conspicuous stimulus features in visual attention. SSP differs from subjective salience estimation, which is based on semantic content and includes reward processing that is altered in ASD [8]. Sensory salience is represented in early sensory processing areas such as the superior colliculi (SC) [9] and the primary visual cortex [10] by a saliency map of visual input that emphasizes sensory distinctive features [3], which are then prioritized in stimulus-driven attention [11]. In ASD, a resting-state hyperconnectivity within these sensory processing areas has been reported [12, 13]. A meta-analysis in ASD further reported elevated activity in the visual cortex during non-social visual processing [14]. These neuroimaging findings indicate increased processing of sensory salience in ASD, irrespective of semantic content [12], which may explain pronounced attention to sensory distinctive features [15] and clinical symptoms of altered sensory reactivity [16].

The locus coeruleus–norepinephrine (LC-NE) system mediates SSP [17] among other mechanisms of neuromodulation [18, 19]. The LC-NE stands out [20] by the ability to increase the signal-to-noise ratio in synaptic signal transmission [21] via transient activity spikes (phasic activity) that occur in response to salience [17, 22]. This LC-NE reactivity to salience represents a neurophysiological filter mechanism of sensory selectivity in early sensory processing [23], which has been explained by connectivity of the pontine LC with the SC [24] and cortical networks of salience estimation [22, 25]. The LC-NE phasic activity releases norepinephrine in sensory processing areas [20, 26] and, thus, emphasizes the sensory processing of salient stimuli [27, 28].

This subcortical mediation of SSP by LC-NE phasic activity has been suggested to underlie stimulus-driven visual attention [29, 30]. In ASD, altered stimulus-driven attention may correspond to an impaired attention disengagement [31] and a slower identification of global versus local information [32]. Thus, we have proposed altered LC-NE phasic activity in SSP as a key mechanism of different visual attentions in ASD that might perpetuate to attenuated gazes on human faces [33].

LC-NE phasic activity is indexed by changes in pupil size [34–36] when controlled for luminance adaptation [37]. While this pupil size is not a continuous activity readout, spikes in activity characterize LC-NE phasic activity and are reflected in pupillary responses [38, 39]. Previous studies in ASD have shown attenuated pupillary responses for targets in neuropsychological [40, 41] and social-cognitive tasks [42, 43], as well as elevated pupillary responses during visuospatial tasks [44, 45]. The visual stimuli in these different tasks share an inherent utility for performance, i.e., they represent semantic content. Thus, these contrasting findings of pupillary reactivity in ASD might be associated with an attenuated adaptation of the LC-NE activity in ASD [46] that results in diverging sensory reactivity to different semantic contents. Recently, an attenuated sensory reactivity has been reported in autistic children, where attenuated pupillary reactivity to oddball stimuli likely indexes a reduced LC-NE phasic activity to semantic content [47, 48]. However, no previous study has explored pupillary reactivity in response to sensory salience, which might alternatively explain diverging sensory reactivity to visual stimuli in ASD.

The sensory salience of stimuli can be quantified by using computer vision approaches that aim to reconstruct human sensory processing in the visual cortex [3]. Algorithms convert input stimuli into an output that highlights sensory conspicuous features (e.g., high contrast) and, thus, approximate saliency maps in human sensory processing [49]. In a seminal paper, these algorithms were applied to extract the sensory salience of naturalistic images that were presented to adult participants [15]. Subsequent gaze analysis showed that the visual attention in autistic adults compared to neurotypical controls was driven more by sensory salience than by semantic content. This supports an increased SSP in ASD [12], although a direct measure of reactivity to sensory salience was missing. Pupillary responses as an index of LC-NE phasic activity can provide such a reactivity measure of SSP. This requires relating pupillary responses to saliency maps in the gaze analysis of visual attention.

Visual attention has often been investigated by assessing gazes on static images [50]. However, static images insufficiently represent naturalistic gaze behavior in dynamic visual attention [51]. In meta-analyses on ASD compared to neurotypical development (TD), gazes on eyes and faces are attenuated [6, 52], while the attenuation is emphasized for dynamic stimuli such as naturalistic videos [51, 53]. In these gaze analyses, relevant semantic content is usually predefined by areas of interest (AOI) [54]. The combination of computer-generated saliency maps and AOI would allow one to quantify the association of sensory salience and semantic content in naturalistic visual attention.

Sensory salience is not independent of semantic content [55, 56]. SSP is also reactive to semantic content such as reward or faces [57]. Thus, LC-NE phasic activity is likely to be evoked by sensory salience and semantic content, which is supported by pupillary responses to sensory [17] as well as semantic [58] aspects. However, an evaluation of semantic content requires higher-order processing and is expected to be slower than the subcortical SSP [59]. This difference in processing time has been utilized to dissociate neurophysiological reactivity into sensory salience and sematic content by investigating the temporal dynamics of pupillary responses [60, 61].

We want to explore SSP as a determinant of social attention, which is described by gazes on semantic content like human faces. The study assesses autistic individuals compared to neurotypical controls during the visual exploration of video scenes with humans and without humans. We estimate SSP by (1) pupillary responses as an index of LC-NE phasic activity and (2) gaze analysis of computer-generated saliency maps for sensory salience. These estimates of SPP are investigated between groups and associated with gazes on human faces. In addition, temporal dynamics will be considered by modeling time and dissecting the pupillary responses into temporal components. We hypothesize [A] an association of pupillary responses to and gazes on sensory salience across groups to establish pupillary responses as an index of reactivity to sensory salience. We further hypothesize [B] group differences in pupillary responses to and gazes on sensory salience to establish different SSP in ASD during visual exploration. Lastly, we hypothesize [C] attenuated gazes on faces in ASD that is moderated by SSP. Collectively, this would support LC-NE phasic activity as a shared underlying mechanism of altered SSP and attenuated social attention in ASD.

## Methods

### Sample

**Fig. 1:**
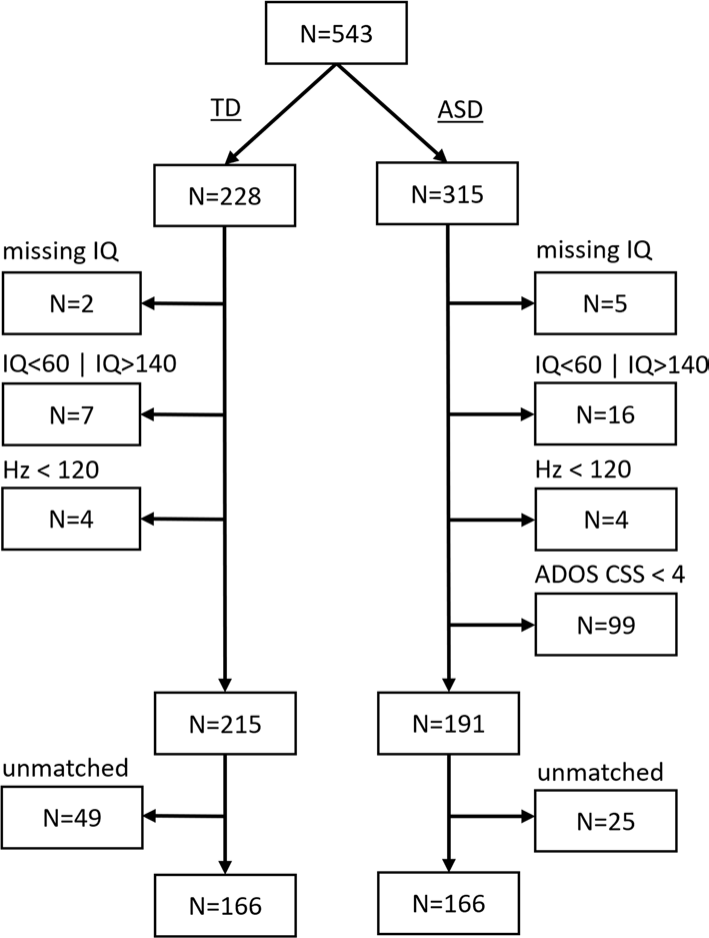
Sample flowchart. The raw data of *n* = 543 were available. We excluded data at the participant level, based on the depicted criteria in Methods. ASD = Autism spectrum disorder, TD = neurotypical controls, Hz = eye-tracker sampling rate, ADOS CSS = Autism Diagnostic Observation Schedule (ADOS-2) Calibrated Severity Score

We included *n* = 166 ASD participants and *n* = 166 matched neurotypical controls (TD) aged 6 to 30 (Table 1). The sample is a subsample of the project “European Autism Interventions—A Multicentre Study for Developing New Medications (EU-AIMS) Longitudinal European Autism Project (LEAP) study” [62] of participants with sufficient eye-tracking data (73.6%). We excluded participants (see Fig. 1) with missing demographic data, a low sampling rate likely due to a false eye-tracker configuration (Hz < 120), particularly low or high IQ scores (IQ < 60 | IQ > 140), and ASD participants with an Autism Diagnostic Observation Schedule (Second Edition; ADOS-2) Calibrated Severity Score (CSS) below the clinical cutoff (CSS < 4) [63]. Groups were matched based on initial group differences in perceptual IQ (i.e., a non-verbal IQ estimate based on the block design and matrix reasoning subtests of the age-appropriate Wechsler Intelligence Scale; WAIS-IV, [64]; WISC-IV, [65]), chronological age, and data quality (eye-tracking accuracy and precision, available data) by the nearest neighbor method with 0.4 SD tolerance.

**Table 1:** Sample description

	ASD	TD	Group difference p-value
N	166	166	–
Age (in years)	15.96/5.59	16.65/6.03	0.280
Sex (female/male)	41/125	49/117	0.387
Perceptual IQ	102.1/17.91	104.81/16.56	0.153
Absolute pupil size (mm)	3.69/0.54	3.63/0.48	0.255
Mean fixation duration (ms)	341.54/40.55	338.92/39.05	0.551
Total screen attention (*s*)	72.41/41.65	77.6/42.86	0.264
Center deviation of gaze (*z*)	-0.05/0.99	0.05/1.01	0.409
Eye-tracking data accuracy (*z*)	0.05/1.05	-0.05/0.94	0.335
Eye-tracking data precision (*z*)	-0.03/1	0.03/1	0.649
Eye-tracking available data (%)	28.2/16.23	30.23/16.7	0.264
Eye-tracking sampling rate (120 Hz/300 Hz)	122/44	134/32	0.151
SRS score	96.85/29.91	28/20.5	< 0.001
ADHD inattention score*	4.35/3.2	1.22/1.94	< 0.001
Anxiety symptoms score*	15.04/11.38	6.89/6.22	< 0.001
Depressive symptoms score*	14.63/12.37	5.88/6.38	< 0.001

Total screen attention is the cumulated duration of fixations in seconds that are considered in the statistical analysis. Eye-tracking data accuracy is the Euclidean distance of gaze estimates and target location, whereas eye-tracking data precision is the Euclidean distance of gaze estimate coordinates per target location in a postdoc, six-point calibration. Eye-tracking available data are the cumulated duration of identified fixations by total duration of investigated scenes in percent

SRS score, Social Responsiveness Scale total score that is established from the autism screening questionnaire; ADHD inattention score, inattention subscale of the DSM-5 ADHD rating scale; Anxiety symptoms score, Beck’s anxiety inventory (BAI) total score; Depressive symptoms score, Beck’s depression inventory (BDI) total score

*Missing data were imputed (see Statistical analysis)

### Procedure

The EU-AIMS LEAP study assessed longitudinal data of a heterogeneous sample of autistic and neurotypical individuals with clinical characterization, neurocognition, genetics, magnet-resonance imaging, electroencephalography, and eye tracking [66]. The current investigation focused on the baseline eye-tracking assessments, which were carried out at six, site-specific eye-tracking laboratories with luminance that is adapted for optimal eye detection (Lux: *m* = 164, SD = 109). Eye tracking was recorded by remote eye trackers (Tobii T120 or TX300, Stockholm, Sweden) at 120 Hz or 300 Hz with a target distance of 65 cm, while the heads could be moved freely. Differences between sites were controlled for in the statistical models by random intercepts for participant.

We analyzed the presentation of natural scenes, which was part of a larger eye-tracking assessment battery [67]. Nine naturalistic videos were presented without a task. Videos were movie clips and cartoons of two categories: (1) humans in social scenes (labeled: human) or (2) scenes without humans (labeled: non-human, Additional file 1: Table S1). Videos were presented for each participant in a pseudo-random order on 17- or 23-inch displays with a fixed display area of 345 × 259 mm. Videos were displayed with audio, although no speech was involved to ensure comparability across multinational sites.

### Data processing

**Fig. 2:**
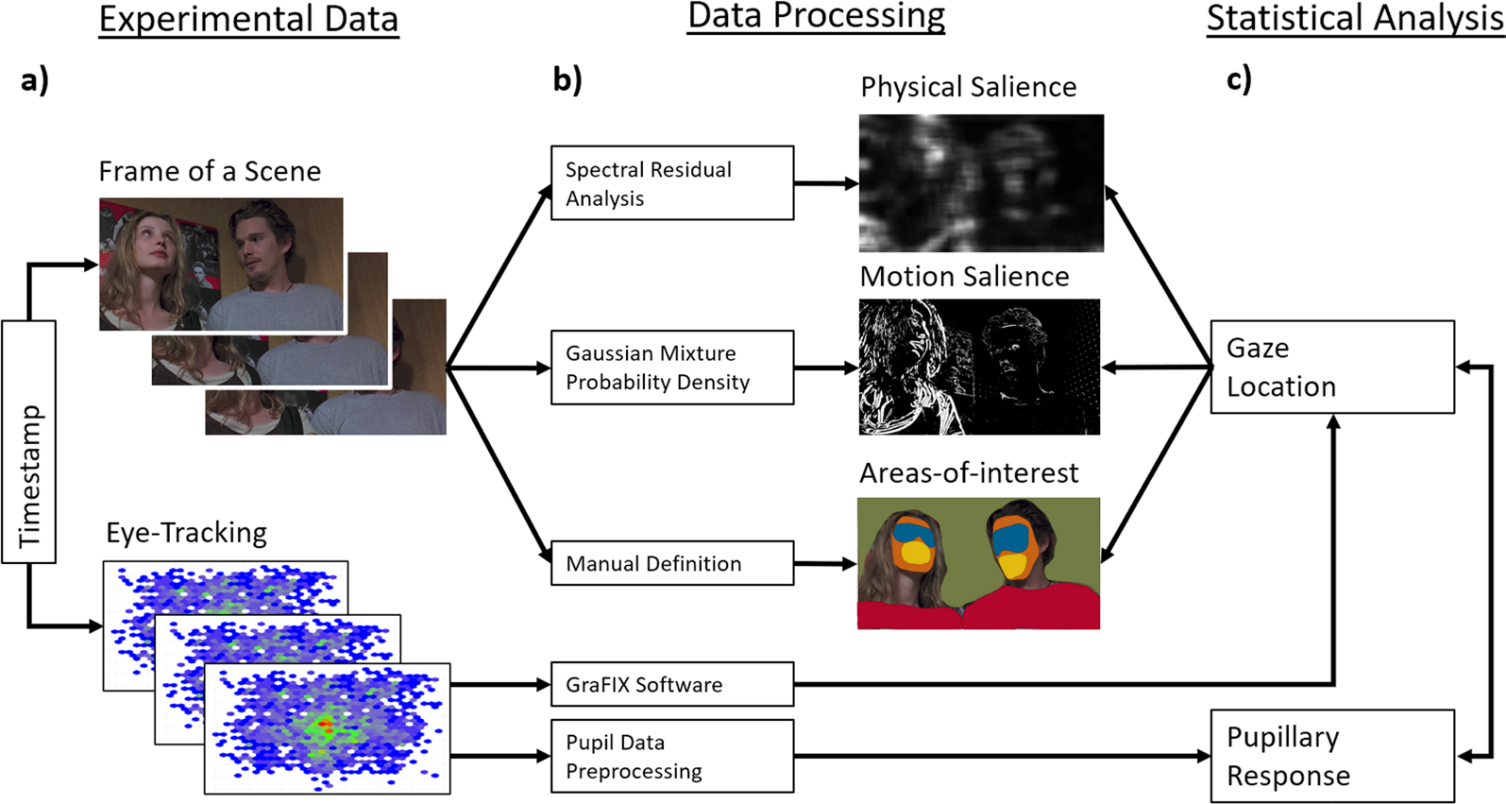
Data processing workflow. Schematic description of the data processing and analysis procedures. **a** Experimental data: Scene frames and eye-tracking raw data were linked by timestamps. **b** Data processing: Scene frames were transformed into two saliency maps by salience estimation algorithms (physical salience, motion salience) and the areas of interest (eyes, mouth, face, body) which were manually defined. Eye-tracking raw data were preprocessed according to recent recommendations [69], and gaze information was estimated by Grafix Software [68]. **c** Statistical analysis: Individual gaze information was matched to both saliency maps and areas of interest and related in the linear mixed models to pupillary reactivity as a proxy of LC-NE phasic activity

A data processing workflow is provided (Fig. 2) and further outlined in the following.

#### Gaze location identification

**Fig. 3:**
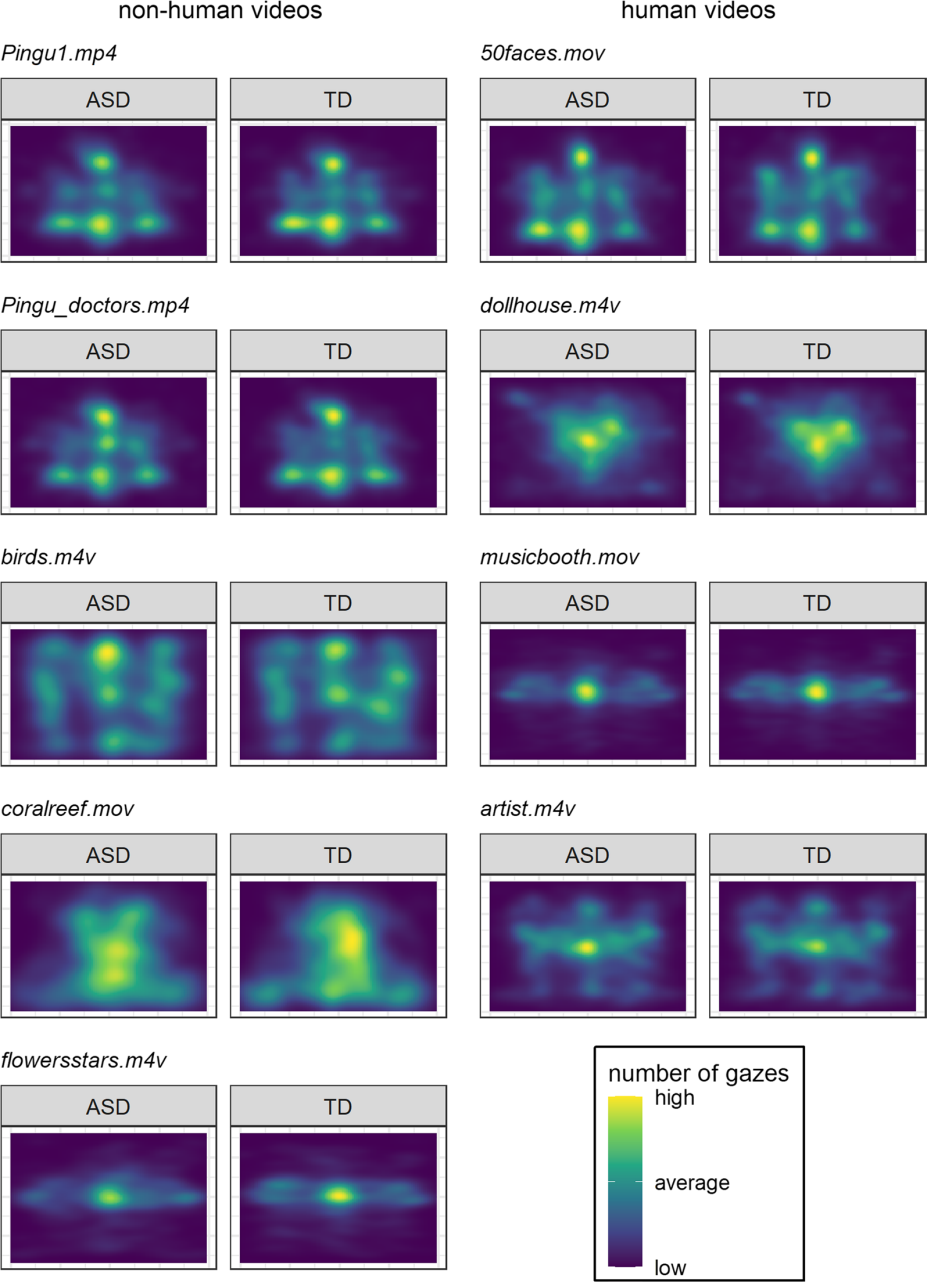
Gaze behavior between groups on the nine naturalistic videos. Gaze behavior represents gaze locations as fixations on the screen that are visualized as heatmaps. Blue colors represent a low number of fixations; green colors represent an average number of fixations; and red colors represent a high number of fixations on the respective video. Of note, the naturalistic videos are segmented to individual scenes in the statistical analysis

In the eye-tracking raw data, gaze events were identified by the Grafix software [68]; for both eyes, raw gaze information data were smoothed temporally (20 samples) and spatially (8 mm). We linearly interpolated missing data up to 100 ms within a displacement threshold of 1° (of the visual angle). Gaze events were differentiated into fixations (i.e., gaze on location) and saccades (i.e., fast eye movements) by a velocity threshold of 20° per second. Consecutive fixations with displacements less than 0.5° were merged. Fixations were only considered valid if they had a duration of longer than 100 ms and a root mean square of less than 1°. Fixations were considered as gaze locations in the present analysis. A comparison of gaze locations for each video (Fig. 3) and gaze behavior metrics (Table 1) suggested comparable gaze behavior between groups.

#### Scene segmentation

We considered visual attention as a dynamic process in the progression of a coherent scene. Thus, we manually segmented the nine naturalistic videos based on camera cuts into a total of 85 scenes. Each scene was assigned to the corresponding video category (human versus non-human). These scenes varied in duration (m/SD = 4001 ms/3543 ms; see Additional file 1: Figure S1) and were chopped in the analysis after 5000 ms to allow for a comparison of the scenes within statistical modeling. We expected that a time scale of 5000 ms would allow for a sufficient interplay of sensory salience and semantic content [60].

#### Pupillary response

**Fig. 4:**
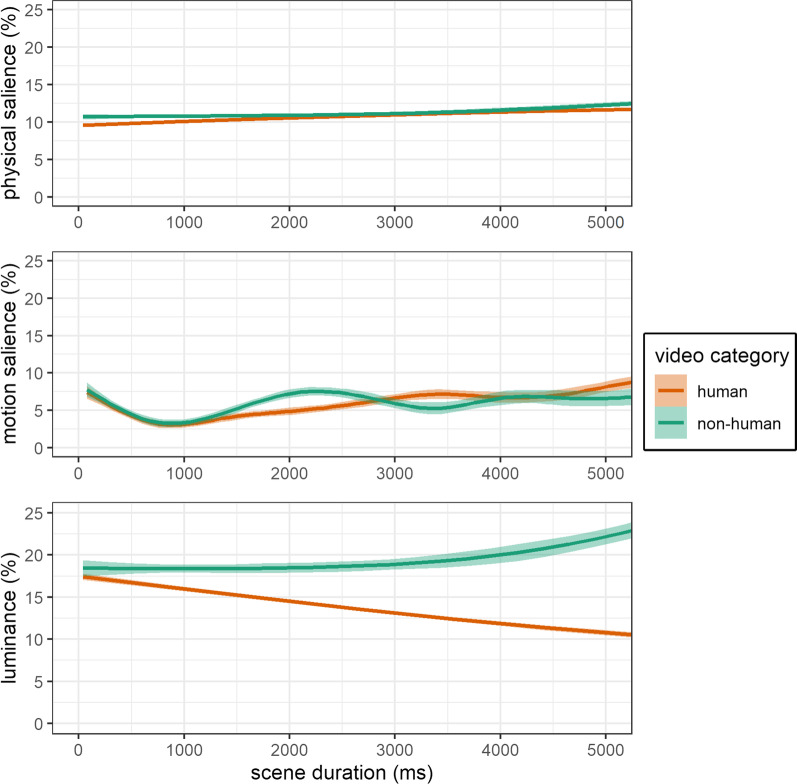
Comparison of physical salience, motion salience and luminance across scenes. The x-axis represents the progression of the video scenes (0–5000 ms), and the y-axis represents the extracted estimates of physical salience (top) or motion salience (middle) or luminance (bottom). The estimates are provided in percentage values of total possible salience or luminance in the current frame. Solid lines represent general additive model fits and shaded areas represent 95% confidence intervals. The physical and motion salience represent overall scene salience estimates of the current frame, which are different to the measures of gazes on physical and gazes on motion salience that represent salience estimates of the current gaze location and are applied in the statistical analysis

Pupil dilation data were provided by the eye-tracking raw data and were preprocessed according to recent recommendations [69]. The onset of a scene did not induce a pupillary light reflex (see Additional file 1: Figure S2). Human scenes were associated with a larger absolute pupil size that is accompanied by a lower luminance in the naturalistic human scenes (see Fig. 4). Pupil size data were standardized by dividing it by each participant’s absolute mean pupil size, while absolute mean pupil size did not differ between groups (Table 1). These standardized pupil dilation data were then aggregated for each gaze location and temporally shifted by 200–400 ms based on the subsequent fixation time point, to account for a delay in pupillary response to presented stimuli [34]. This provided an index of change in pupil size as a measure of relative pupillary response to fixated stimulus features.

We further extracted two temporal components from the preprocessed pupil dilation data by principal component analysis (PCA) with varimax rotation [60]. These two components provided a temporally meaningful loading structure and provided the best fit with the least components (Additional file 1: Figure S3; Additional file 1: Table S2). We multiplied the pupillary response during each fixation based on time by the corresponding loadings on the first or second component; this provided measures of the early-weighted (PR1) and late-weighted (RP2) pupillary responses.

#### Salience estimation

We applied the open-source computer vision library (OpenCV 4.4.0) by custom python scripts (version 3.8) [70] to extract physical and motion salience information that are considered relevant modalities of sensory salience [49]. This is based on Itti and Koch’s computational model of visual attention [3] which aims to generate saliency maps that replicate bottom-up selective sensory processing up to the primary visual cortex [10]. For each sequential video frame, the input data were the RGB matrix (2-D picture information), and the output data were separate grayscale matrices for physical and motion salience. Physical salience was estimated by spectral residual analysis [71] that identifies non-redundant information and has been considered a “fast and effective” estimator of early visual sensory processing [49]. Motion salience was estimated by Gaussian mixture probability density [72] that subtracts spatial background information by comparing arrays of weighted frames (alpha factor = 0.002) and delivers proto-object recognition in sequential data without a priori semantic knowledge [73].

A comparison of physical and motion salience between video scene categories (human versus non-human) is provided below (Fig. 4). Physical salience and motion salience were weakly correlated (*r*(9938)  = 0.11, *p* < 0.001) and thus are considered as distinct features of sensory salience. Physical salience and motion salience were matched to corresponding gaze locations to derive individual estimates of gazes on sensory salience for each video frame.

#### Luminance estimation

We estimated luminance with the RGB matrix of the video frames [74]. RGB values were gamma-corrected, converted to linear scales and transformed to a coefficient of luminance (*L* = 0.2126 * *R* + 0.7152 * *G* + 0.0722 * *B*). Luminance was weakly correlated with physical salience (*r*(9938) = 0.02, *p* = 0.013) and motion salience (*r*(9938) = 0.12, *p* < 0.001) (see Fig. 4). A 2-D convolutional smooth with a Gaussian kernel (sigma = 10, *x* and *y* = 10% of frame size) was applied as a blur filter to estimate a local luminance within video frames. Gaze locations were matched to the corresponding local luminance to derive the luminance of each gaze location.

#### Gazes on faces

We defined the eyes, mouth, face, and body of humans as the areas of interest (AOI). This was achieved by manually drawing in these areas for each video frame (supplemental information S15). AOI were only available in scenes that depicted humans (*k* = 36) and some of these scenes were only presented to adolescent and adult participants (*k* = 21); this was controlled for in the linear mixed models. The proportion of gaze locations within each AOI was applied as an individual estimate of gaze allocation in the progression of a video scene. This estimate is independent of absolute screen attention, which did not differ between groups (see Table 1). We focused our analysis on gazes on faces as the video scenes predominantly consisted of total shots that were not close enough to reliably differentiate between the eyes and mouth AOI. Recent research has emphasized gazes on faces compared to gazes on eyes as a robust group difference between ASD and TD [75].

### Statistical analysis

**Fig. 5:**
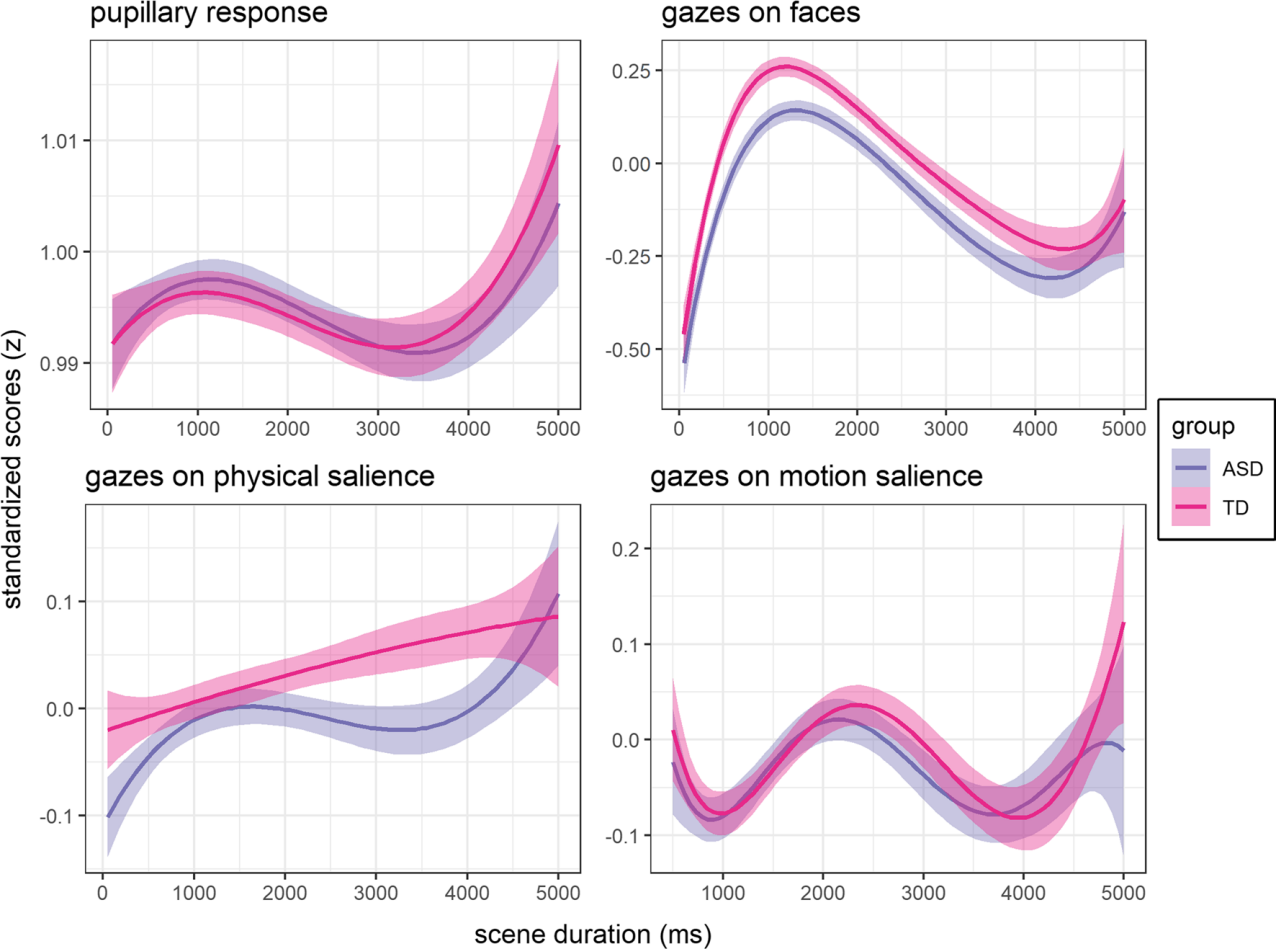
Progression of key variables aggregated across scenes. Magenta: neurotypical controls (TD); purple: individuals with autism spectrum disorder (ASD). Solid lines represent general additive model fits, and shaded areas represent 95% confidence intervals

The statistical analysis was done in R 4.2 including additional packages (see supplemental information S16). The data were analyzed by linear mixed models using restricted maximum likelihood estimation to investigate [A] the association of pupillary response and gazes on physical salience and gazes on motion salience, [B] group differences in these three estimates of SSP, and [C] group differences in gazes on faces (Fig. 5). We further included the pupillary response and gazes on physical and motion salience as independent predictors in the gazes-on-faces model to investigate their moderating effects. We applied group (ASD vs. TD) and video category (human vs. non-human) as fixed effects, whereas the gazes-on-faces model was restricted to the human video scenes. The video category was included as a fixed effect to compare SSP in human versus non-human video scenes.

We included a polynomial fixed effect of time (i.e., scene duration, 0–5000 ms) to consider the temporal dynamics of SSP in the progression of a scene. A third-degree polynomial provided the best and most parsimonious fit in describing changes in pupillary response (Additional file 1: Table S3). We allowed interactions between the fixed effects relevant to the hypotheses (group, video category, time).

We applied random intercepts for participant and video scene to account for the interdependence of measurements. The random intercept for participant further controls for interindividual differences and differences between recruiting sites, while the random intercept for scene further controls for differences in stimulus characteristics between scenes. These random intercepts also control for participant- and trial-specific differences in missing data. We further controlled for various potential confounders by fixed effect covariates for sex, age, perceptual IQ, comorbid psychopathology, data quality, gaze behavior, and luminance (see supplemental information S17). Full model definitions are provided in Additional file 1: Table S4. A more complex random effect structure with a random slope for participant did not lead to converging models and thus was not further considered.

In a secondary analysis, we applied the early and late pupillary response components as alternative fixed effects to investigate the moderating effects on gaze behavior (hypothesis C). This weighted pupillary response comprises temporal information by factor weighting, and thus, the polynomial fixed effect of time was dropped for this analysis.

In a supplementary analysis, we checked whether sensory symptoms as measured by the short sensory profile would be associated with the dependent variables across and between groups. For this, we exchanged the group variable by the short sensory profile total score in all models. We did not observe any significant main effects of sensory symptoms, and thus, it was not further considered.

Fixed effect significance was estimated by ANOVA using Satterthwaite’s method [76]. We adjusted for multiple comparisons (3 analyses) by FDR correction (*p*-adj.). Fixed effects were reported as standardized coefficients (*β*) that represent an effect size. Interactions were investigated post hoc with marginalized means/coefficients (Δ*M*/Δ*β*). Effects are presented with 95% confidence intervals (95% CI). Model comparisons applied refitting with maximum likelihood estimation. The full model results, including all the covariates, are described in the supplements (Additional file 1: Tables S1–S8). Here, we only report the fixed effects that are relevant to the hypotheses (group, video category).

### Power analysis

Power was estimated by simulation based on the observed random effect variance [77]. Confidence intervals were based on 1000 iterations. For small, fixed effects (*β* = 0.1), we achieved a power of 90.1% with the applied linear mixed models, 95% CI [88.1, 91.8].

## Results

### Gazes on sensory salience differed by pupillary response [hypothesis A]

Gazes on physical salience were lower when viewing human videos compared to non-human videos (*F*(1, 302) = 15.17, *p*-adj*.* < 0.001, *β* = − 0.35, 95% CI [− 0.61, − 0.08]). Gazes on physical salience were also lower for higher pupillary responses (*F*(1, 51,986) = 21.19, *p*-adj. < 0.001, *β* = − 0.17, 95% CI [− 0.31, − 0.04]), which differed by video category (*F*(1, 27,988) = 8.734, *p*-adj*.* = 0.009). Post hoc analysis showed that the effect of pupillary response on physical salience was specific to non-human video scenes (Δ*β* = − 0.29, 95% CI [− 0.42, − 0.15]) compared to human video scenes (Δ*β* = − 0.10, 95% CI [− 0.26, 0.06], Additional file 1: Table S5).

**Fig. 6:**
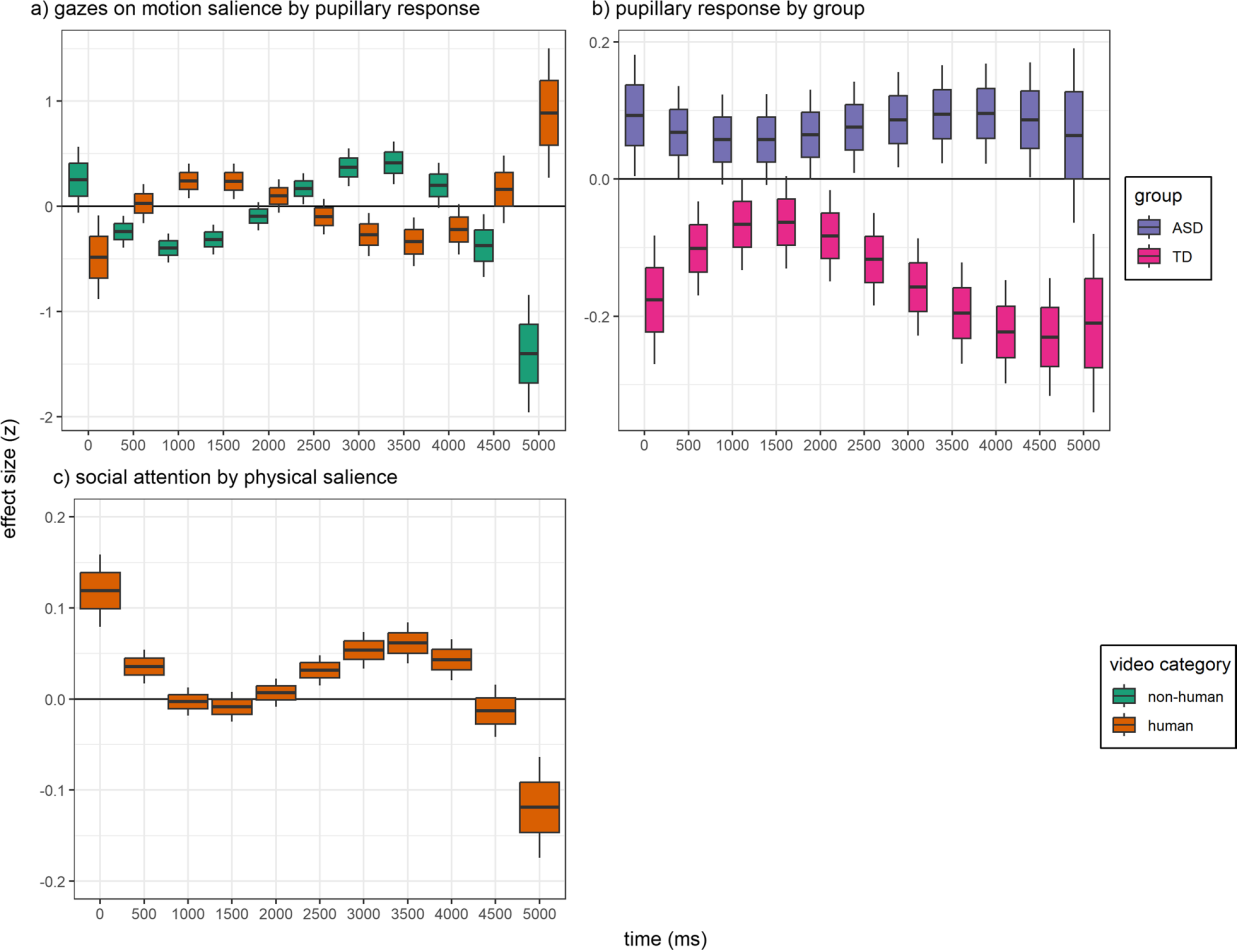
Temporal effects in SSP. The x-axis represents the progression of the video scenes (0–5000 ms), and the y-axis represents the estimated marginalized means of standardized effects. Boxplot whiskers correspond to ± 2 standard errors, and the interquartile range corresponds to ± 1 standard error. Boxplots separated by video category or group are displayed side by side for each time point to avoid overplotting. **a** The effect of pupillary response on gazes on motion salience. **b** The effect of group on pupillary response. Boxplots represent effect size contrasts [ASD-TD]. **c** The effects of gazes on physical salience on social attention

Gazes on motion salience were described by an interaction of video category x pupillary response x time (*F*(1, 66,329) = 53.54, *p*-adj. < 0.001). Post hoc analysis showed that the effect of pupillary response dynamically changed over time (Fig. 6a, Additional file 1: Table S6).

### Pupillary response differed by group and video category [hypothesis B]

The pupillary response was higher when viewing human compared to non-human video scenes (*F*(1, 82) = 20.01, *p-adj.* < 0.001, *β* = 0.39, 95% CI [0.18, 0.60]), which differed between groups (*F*(1, 66,329) = 304.54, *p*-adj. < 0.001). Post hoc analysis in ASD compared to TD showed that the pupillary response was higher for non-human videos (Δ*M* = 0.06, 95% CI [0.00, 0.13]), while it was lower for human videos (Δ*M* = − 0.08, 95% CI [− 0.14, − 0.01]); this interaction differed further with time, as indicated by a three-way interaction of group x video category x time (*F*(1, 66,056) = 6.43, *p*-adj*.* = 0.033). Post hoc analysis indicated that differences in the pupillary response between groups for human videos were attenuated in the early phases of the scenes (1000–2000 ms, see Additional file 1: Table S7, Fig. 6b).

### Group differences in gazes on faces were influenced by gazes on physical salience [hypothesis C]

**Fig. 7:**
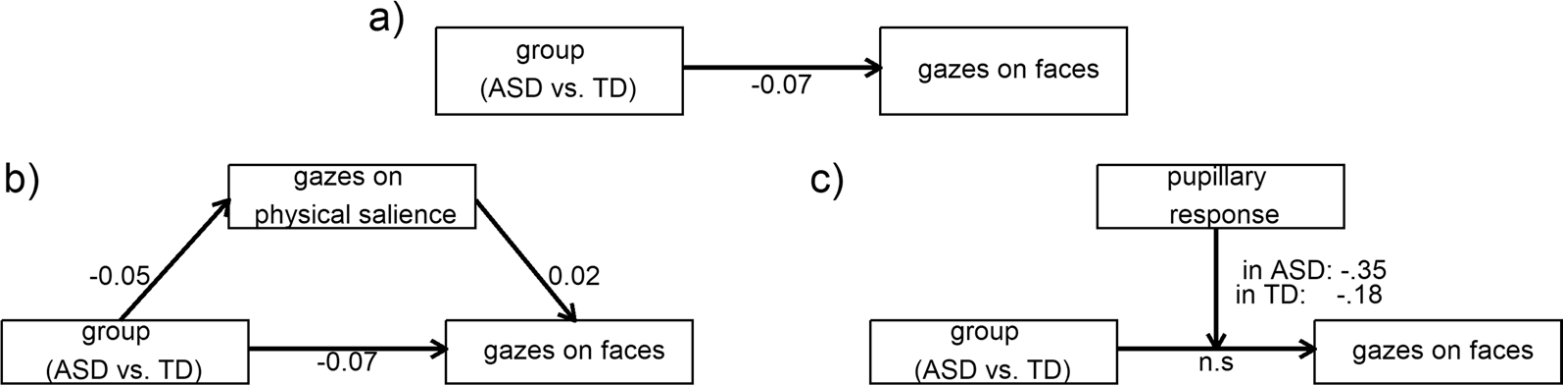
Group differences in gazes on faces are modulated by SSP. **a** Group difference in gazes on faces between ASD and TD as index of social attention. **b** Gazes on physical salience are associated with the group difference in gazes on faces. **c** Pupillary response moderated the group difference in gazes on faces. n.s. = not significant

Gazes on faces were indicated to be lower in ASD compared to TD (*F*(1, 290) = 5.08, *p*-adj*.* = 0.075, *β* = − 0.07, 95% CI [− 0.14, − 0.01], Additional file 1: Table S8, Fig. 7a). The group effect was emphasized when excluding all covariates (*F*(1, 298) = 17.29, *p*-adj < 0.001, *β* = − 0.12, 95% CI [− 0.18, − 0.06], Additional file 1: Table S9).

The inclusion of physical salience as fixed effect delivered a better model fit (*χ*^2^(8) = 57.68, *p* < 0.001, Additional file 1: Table S10). The main effect of physical salience (*F*(1, 30,175 = 15.28, *p*-adj*.* < 0.001) was a positive association with gazes on faces across groups (*β* = 0.02, 95% CI [0.01, 0.04]). In secondary analyses, gazes on physical salience (*F*(1, 223) = 8.58, *p*-adj. = 0.012, Δ*M* = − 0.05, 95% CI [− 0.08, − 0.02]) were lower in ASD compared to TD for human scenes (Additional file 1: Table S11). Together, this indicated that gazes on physical salience could mediate the group difference in gazes on faces (Fig. 7b).

Gazes on physical salience further differed by time (*F*(1, 30,125) = 42.46, *p*-adj*.* < 0.001). Post hoc analysis indicated that the gaze effect of physical salience on faces was attenuated when the mean gazes on faces was at its maximum during the video clips (1000–2000 ms, see Additional file 1: Table S10, Fig. 6c). There were no group differences in gazes on motion salience for human scenes (*F*(1, 207) = 0.12, *p*-adj. = 1, Δ*M* = 0, 95% CI [− 0.03, 0.02]) (Additional file 1: Table S12), and thus, the effect of motion salience as a mediator was not further investigated.

We further observed a covariate effect of age on gazes on faces (*F*(1, 294) = 24.62, *p*-adj. < 0.001), which was not altered by the inclusion of physical salience or pupillary response as fixed effect. A higher age was associated with higher gazes on faces (*β* = 0.07, 95% CI [0.04, 0.10]).

### Group differences in gazes on faces were moderated by a higher early pupillary response [hypothesis C]

The inclusion of the pupillary response as an alternative fixed effect rendered the group difference in gazes on faces to be non-significant (*F*(1, 12,006) = 2.29, *p*-adj*.* = 0.390; Additional file 1: Table S13) and delivered a better model fit (*χ*^2^(8) = 25.56, *p* < 0.001). A full moderation is indicated by the main effect of pupillary response (*F*(1, 28,786 = 6.29, *p*-adj*.* < 0.036) that was a negative association with gazes on faces across groups (*β* = − 0.24, 95% CI [− 0.09, − 0.38]). Post hoc analysis showed that the effect of pupillary response might be more pronounced in ASD (Δ*β* = − 0.35, 95% CI [− 0.56, − 0.15]) compared to TD (Δ*β* = − 0.18, 95% CI [− 0.35, − 0.01], Fig. 7c).

In a secondary analysis, we investigated whether the group difference in the effect of pupillary response on gazes on faces was because of early or late pupillary responses. We, thus, exchanged the pupillary response variable by the early (PR1) and late (PR2) pupillary response components (Additional file 1: Table S14). This inclusion also rendered the group difference in gazes on faces to be non-significant (*F*(1, 296) = 4.03, *p*-adj. = 0.138). Interestingly, PR1 was negatively associated with gazes on faces (*F*(1, 30,189) = 38.92, *p*-adj*.* < 0.001, *β* = − 0.04, 95% CI [− 0.05, − 0.02]), while PR2 was positively associated with gazes on faces (*F*(1, 30,261) = 16.44, *p*-adj*.* < 0.001, *β* = 0.01, 95% CI [0.00, 0.03]).

## Discussion

We provide the first empirical study that relates sensory salience processing (SSP) to gazes on faces during naturalistic visual attention in a comprehensive sample of autistic individuals and neurotypical controls. We utilized computer vision algorithms to derive estimates of gazes on sensory salience, while we applied the pupillary response as a proxy of LC-NE phasic activity to assess neurophysiological reactivity to sensory salience. Gazes on and reactivity to sensory salience were associated while sensory reactivity differed between groups. As a main finding, reactivity to sensory salience moderated attenuated gazes on faces in ASD. The findings indicate that altered SSP is associated with an attenuated social attention in ASD.

### Sensory salience processing in naturalistic visual attention across groups

Gazes on physical salience were lower in video scenes with humans compared to scenes without humans, although overall physical salience is similar between the video categories (Fig. 4). This is in line with a previous study in neurotypical adults and could be explained by a superior semantic salience of displayed humans that is prioritized in visual exploration compared to physical salience. Across scenes, gazes on physical salience did not differ between ASD and TD, which corresponds to previous findings for static images [78]. In a secondary analysis for human videos, we found lower gazes on physical salience in ASD compared to TD. Both findings contrast the seminal study by Wang et al. [15] that reported increased physical salience for static images in autistic adults. Their conclusion was derived from a machine learning analysis that compared gaze behavior between smaller groups (ASD: *n* = 20; TD: *n* = 19); thus, the small sample size may have limited the generalizability of their findings. Our findings suggest that attention to sensory salience is described by attenuated gazes on physical salience in the visual exploration of dynamic stimuli depicting humans, which could be specific to ASD.

We examined motion salience as an additional dimension of gazes on sensory salience, which is understudied in visual exploration. In our study, gazes on motion salience did not differ between ASD and TD. This contrasts a previous study with a smaller sample (ASD: *n* = 26; TD: *n* = 15) of preschool children [79], which reported decreased gazes on motion salience in ASD for social videos. Our well-powered sample supports the conclusion that gazes on motion salience, as well as physical salience, are both proxies of attention to sensory salience during naturalistic visual exploration [3].

Pupillary responses were associated with these proxies of attention to sensory salience. This association was negative for physical salience across scenes, whereas, for motion salience, it was negative in the early phases (500–1500 ms) and positive in the intermediate phases (2500–4000 ms) of the non-human scenes, whereas this pattern inversed for human scenes (Fig. 6a). These findings may indicate distinct mechanisms that influence SSP and are reflected in dynamic LC-NE phasic activity [80]. We speculate that semantic content, such as conspicuous objects, causes a top-down salience response that induces LC-NE phasic activity [22] and overrides a bottom-up attention to sensory salience [3] that is rather prevalent during the intermediate phase of a scene. This dynamic reactivity to sensory salience warrants further investigation.

### Pupillary responses indicate different sensory salience processing in ASD

For human scenes, pupillary responses were decreased in ASD compared to TD. This corresponds to previous findings that reported attenuated pupillary responses for social versus non-social images [81], insensitivity of pupillary responses to changes in the intensity of social content [43], and attenuated pupillary responses to social-emotional content [42]. In these studies, the pupillary responses may have reflected the subjective utility of the presented stimuli (i.e., semantic content) that is associated with LC-NE phasic upregulation in order to emphasize the sensory processing of the salient stimuli [21]. Thus, our findings extend earlier reports of decreased pupillary responses in ASD to naturalistic movie scenes and indicate a reduced LC-NE phasic activity in response to human stimuli. The group difference was attenuated when mean gazes on faces were highest across the groups (1000–2000 ms, see Fig. 6b). In ASD, gazes on faces may alleviate, but do not compensate for, attenuated LC-NE phasic activity in response to human stimuli.

For non-human scenes, pupillary responses were increased in ASD compared to TD. This could relate to previous findings of increased pupillary responses for a non-human visual search task in toddlers [45] or in response to unexpected targets during a visuospatial reaction time task in adolescents [44]. This may indicate increased sensory salience processing in ASD for non-human stimuli that can lead to superior performance in associated tasks [82]. Taken together, our pupillometric findings in ASD could suggest a bias in SSP that prioritizes non-human over human content in visual exploration. This may further explain gaze preferences for geometric over human stimuli during concurrent presentation, which has been established as a prognostic marker of ASD [83].

### Attenuated gazes on faces are moderated by sensory salience processing

In human scenes, gazes on faces were lower in ASD compared to TD. This corresponds to meta-analytic findings of attenuated social attention [6, 53]. As a main finding of the current study, attenuated gazes on faces in ASD (a.) might be mediated by effects of gazes on physical salience across groups and (b.) were moderated by pupillary reactivity within ASD. Across groups, gazes on physical salience were associated with gazes on faces, while gazes on physical salience were lower in ASD compared to TD. We further observed that higher age was associated with more gazes on faces, which was independent from the mediation and moderation effects. Our findings provide evidence that decreased attention to physical salience might correspond to lower social attention in ASD and TD (Fig. 7B mediation).

In addition, higher pupillary responses were associated with substantially lower gazes on faces, which might be more pronounced in ASD. This was characterized further by our secondary analysis that showed a negative association of an early pupillary response component (PR1), but a positive association of a late pupillary response component (PR2) with gazes on faces. Based on previous research on fast and slow pathways of visual processing [59] and on the reactivity of the salience network to sensory salience and semantic content [55, 56], PR1 may overlap with the LC-NE phasic reactivity to sensory salience, while PR2 may overlap with the LC-NE phasic reactivity to semantic content. Thus, in ASD, we conclude that increased LC-NE phasic reactivity to sensory salience is associated with lower social attention. This supports an earlier conceptualization of an altered “fast-track” processing of sensory information that underlies atypical eye contact in ASD [84]. In addition, higher early pupillary responses in ASD have been related to an over-responsivity to sensory changes [41]. Our findings relate these early pupillary responses as an index of sensory processing to an attenuated social attention. Altered LC-NE phasic activity may provide an underlying mechanism to relate ASD clinical phenotypes of altered sensory processing to atypical social attention.

In addition, increased PR2 was associated with increased gazes on faces which could, therefore, provide a compensating mechanism in ASD. The diametrically opposite effects of the early (PR1) and late (PR2) pupillary responses on gazes on faces underline the temporal dynamics of LC-NE phasic activity in moderating visual attention that may reflect distinct underlying mechanisms [80]. Independent studies need to replicate early and late LC-NE phasic activity as differential moderators of social attention.

## Limitations

Our naturalistic study design precluded experimental manipulations and comprised that stimulus salience and luminance are not controlled across scenes. The heterogeneous sample may have led to reported effect sizes that are only small to moderate. Accordingly, the significant covariate effects of age and perceptual IQ on social attention indicate that the reported effects may differ between specific developmental subgroups. The exclusion of participants with an IQ < 60 led to findings that are not generalizable to individuals with moderate to severe intellectual disabilities. Lastly, numerous cognitive processes and functional pathways induce LC-NE phasic activity, and we are not able to differentiate those [24, 85].

## Conclusions

We characterize sensory salience processing in visual attention. Findings emphasize a moderating role of LC-NE phasic activity in gazes on sensory salience and suggest an increased reactivity to sensory salience as a bottom-up mechanism of attenuated gazes on faces in ASD. Altered LC-NE phasic activity may represent a neurophysiological mechanism of altered sensory salience processing that underlies the clinical phenotypes of altered sensory processing and attenuated social attention.

## Supplementary Information

**Additional file 1**. Further information on duration of video scenes, pupil size comparisons between groups and video category, pupillary response components, stimuli characterization, comparison of model fits, model definitions, full linear mixed model results, Area-of-interest (AOI) definition criteria, applied R packages, and covariates in the statistical analyses.

## Data Availability

The datasets generated and/or analyzed during the current study are not publicly available due to an embargo period but are available from the corresponding author on reasonable request. The data analysis scripts are accessible online (https://github.com/nicobast/project_video_salience).

## Abbreviations

AOI: Areas of interest
ADOS-2: Autism Diagnostic Observation Schedule, Second Edition
ASD: Autism spectrum disorder
CSS: Calibrated severity score
RP1: Early pupillary response component
RP2: Late pupillary response component
LC-NE: Locus coeruleus–norepinephrine system
MICE: Multiple imputations of chained equations
TD: Neurotypical development
PCA: Principal component analysis
SSP: Sensory salience processing
SC: Superior colliculi
