# Supplementary material for: Sensory salience processing moderates attenuated gazes on faces in autism spectrum disorder: a case–control study
Source: Mol Autism. 2023 Feb 9;14:5. doi: 10.1186/s13229-023-00537-6 (PMC9912590; doi:10.1186/s13229-023-00537-6)
Supplement: Supplementary file 1 — Additional file 1. Further information on duration of video scenes, pupil size comparisons between groups and video category, pupillary response components, stimuli characterization, comparison of model fits, model definitions, full linear mixed model results, Area-of-interest (AOI) definition criteria, applied R packages, and covariates in the statistical analyses. [file 13229_2023_537_MOESM1_ESM.docx]

**Table of Content**

[*Figure S1*. Duration of individual video scenes. 2](#_Toc106808636)

[*Figure S2.* Absolute pupil size comparison between groups and video category. 3](#_Toc106808637)

[*Figure S3.* Pupillary response components. 4](#_Toc106808638)

[*Table S1.* Stimuli Characterization. 5](#_Toc106808639)

[*Table S2*. Comparison of model fits: pupillary components 7](#_Toc106808640)

[*Table S3*. Comparison of polynomial fits: pupillary response. 8](#_Toc106808641)

[*Table S4*. Model definitions and syntax. 9](#_Toc106808642)

[*Table S5*. Linear mixed model: physical salience. 10](#_Toc106808643)

[*Table S6*. Linear mixed model: motion salience. 11](#_Toc106808644)

[*Table S7*. Linear mixed model: pupillary response. 13](#_Toc106808645)

[*Table S8*. Linear mixed model: social attention by group. 14](#_Toc106808646)

[*Table S9*. Linear mixed model: social attention by group without mediators and covariates. 15](#_Toc106808647)

[*Table S10.* Linear mixed model: social attention with gazes on physical salience as mediator. 16](#_Toc106808648)

[*Table S11*. Linear mixed model: gazes on physical salience associated with group. 17](#_Toc106808649)

[*Table S12*. Linear mixed model: motion salience associated with group. 18](#_Toc106808650)

[*Table S13*. Linear mixed model: social attention by group with pupillary response as moderator. 19](#_Toc106808651)

[*Table S14*. Linear mixed model: social attention by group with pupillary components as moderators 20](#_Toc106808652)

[*Supplemental Information S15.* Areas-of-interest (AOI) definition criteria. 21](#_Toc106808653)

[*Supplemental Information S16.* Citations of applied R packages 22](#_Toc106808654)

[*Supplemental Information S17.* Covariates in the statistical analyses. 25](#_Toc106808655)

# *Figure S1*. Duration of individual video scenes.


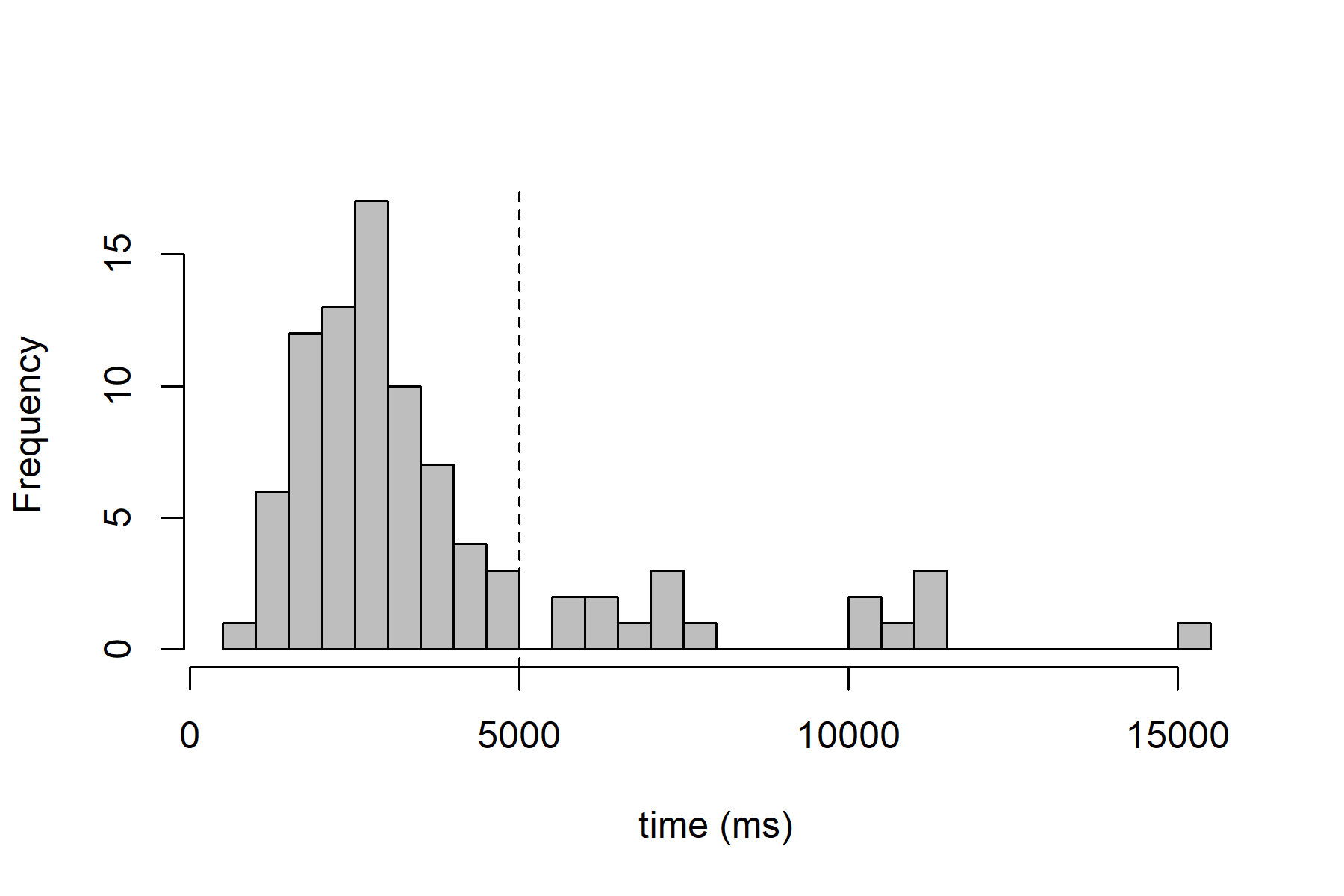


*Note*. Vertical line represents the cutoff for inclusion as data in the statistical models.

# *Figure S2.* Absolute pupil size comparison between groups and video category.


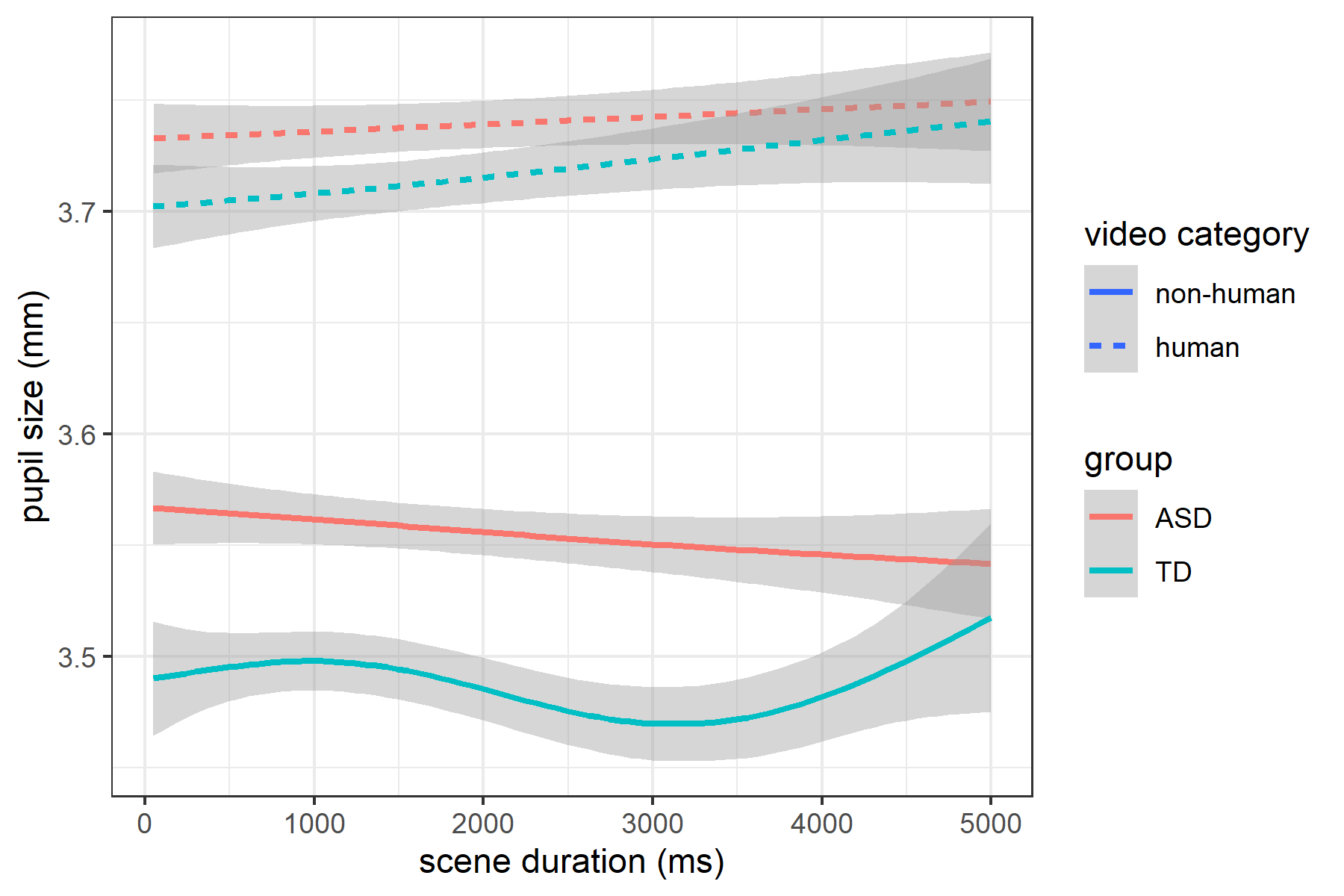


*Note*. Line color represents the different groups, while line type represents the different video categories.

# *Figure S3.* Pupillary response components.


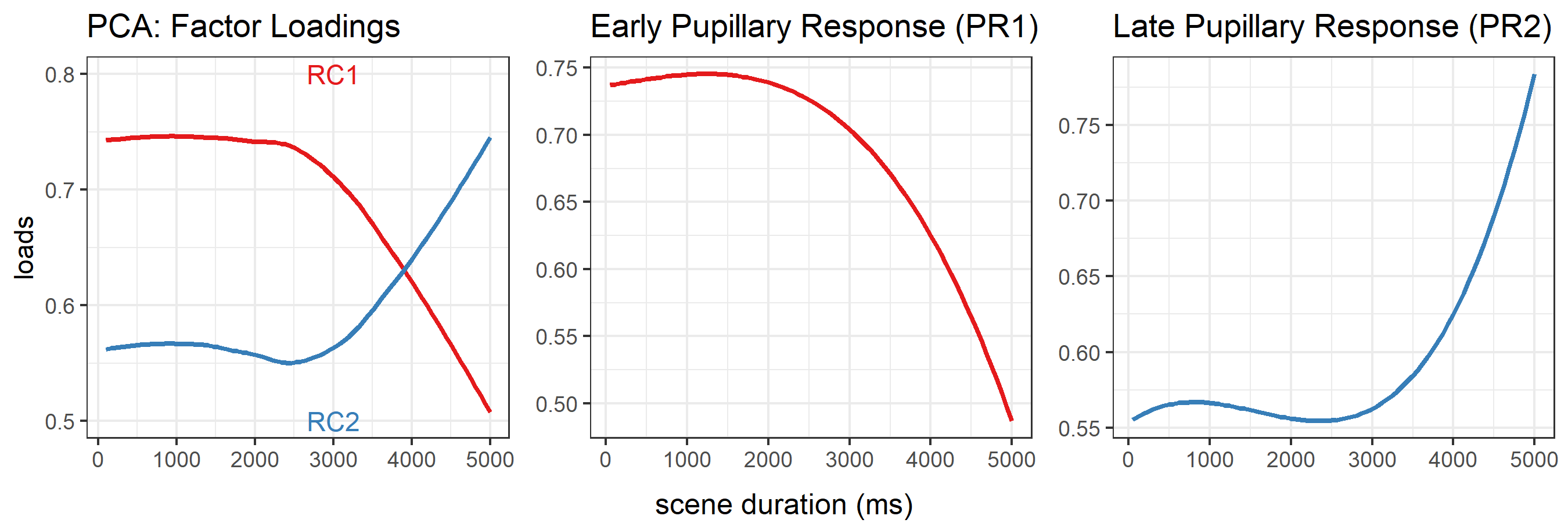


*Note*. Left: Factor loadings of the two-component solution in the principal component analysis of pupillary responses. Individual pupillary responses were multiplied by the respective factor loadings to retrieve a measure of early-weighted pupillary response (PR1, middle) and late-weighted pupillary response (PR2, right).

# *Table S1.* Stimuli Characterization.

*Download Link:* [*https://hessenbox-a10.rz.uni-frankfurt.de/dl/fi2rRR2ceLHc2dSPrin1tLx3*](https://hessenbox-a10.rz.uni-frankfurt.de/dl/fi2rRR2ceLHc2dSPrin1tLx3)

|  | *description* | *sample frame* |
| --- | --- | --- |
| **human videos:** |  |  |
| *50faces.mov* | Different people are interviewed on the street. Mainly close-up faces | 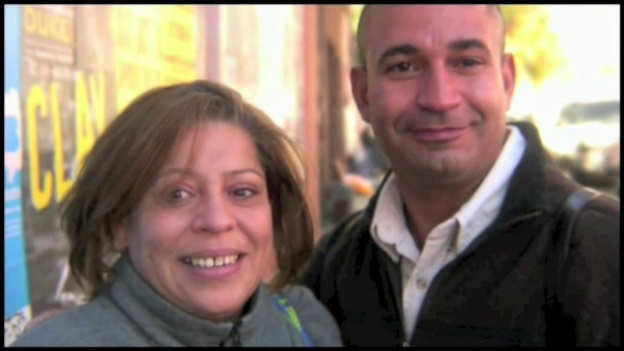 |
| *artist.m4v* | An excited crowd cheers the protagonist. Scene from the movie “The Artist (2012)” | 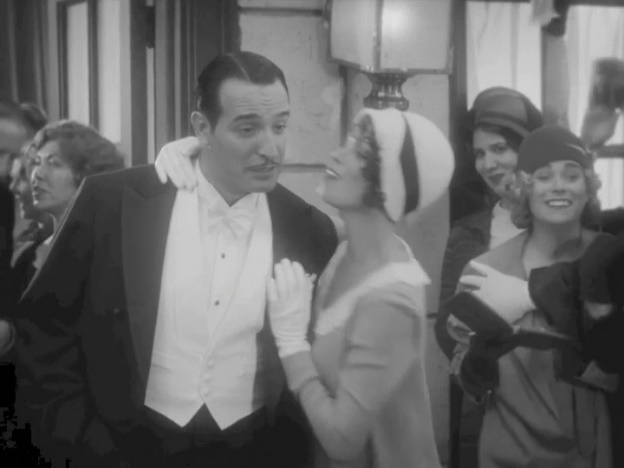 |
| *dollhouse*.m4v | A teenage girl tries to find a spot in the school cafeteria and interacts with another girl –Scene from the movie “Welcome to the dollhouse (1995)” | 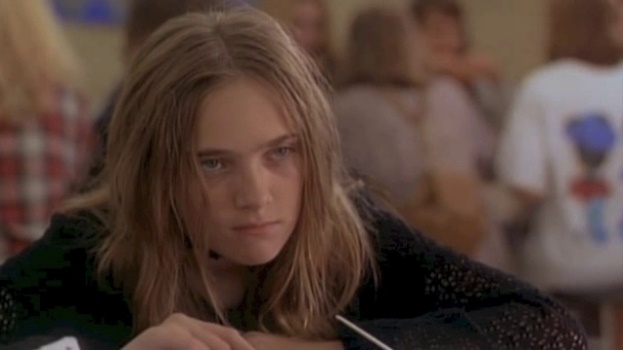 |
| *musicbooth*.mov | A man and a woman are nonverbally interacting with each other while listening to a song. Scene from the movie “Before Sunrise (1995)” | 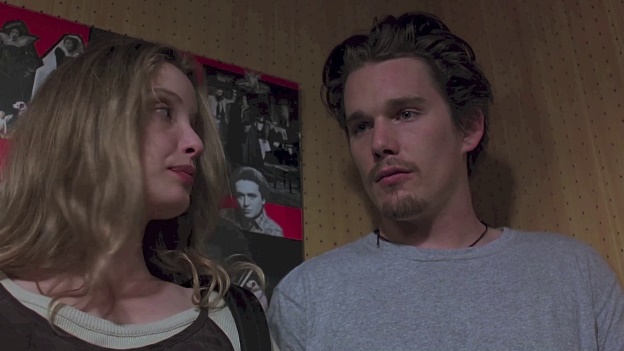 |

| **non-human videos:** |  |  |
| --- | --- | --- |
| *birds.m4v* | various bird species are moving on tree branches | 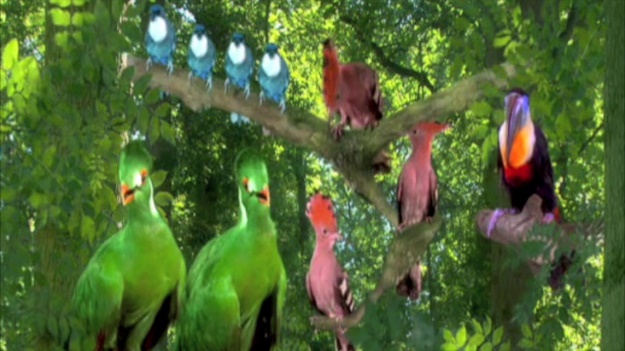 |
| *coralreef.mov* | scenic camera ride over a coral reef with a small plane | 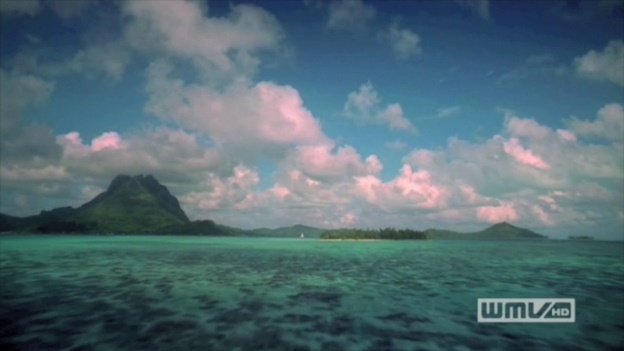 |
| *flowerstaars.m4v* | A spangled sky turns into a sea of flowers | 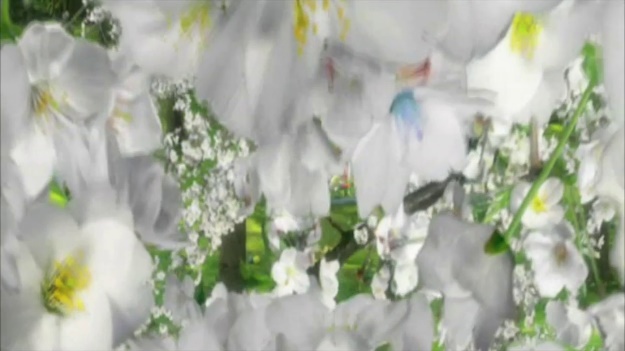 |
| *pingu1.mp4* | Two cartoon penguins write together on a slate | 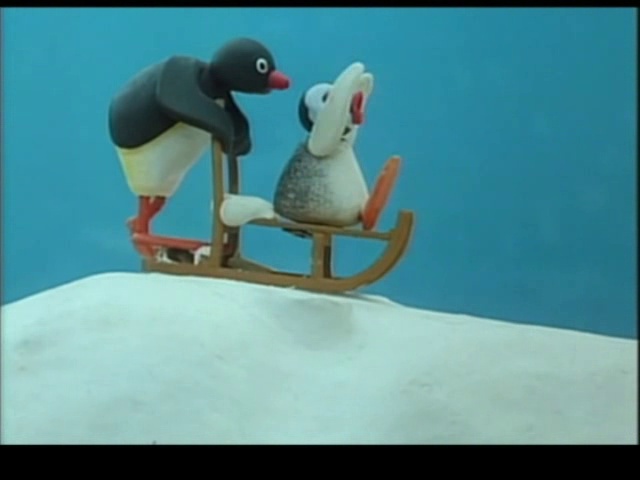 |
| *pingu_doctors.m4v* | Two cartoon penguins are playing together in a house | 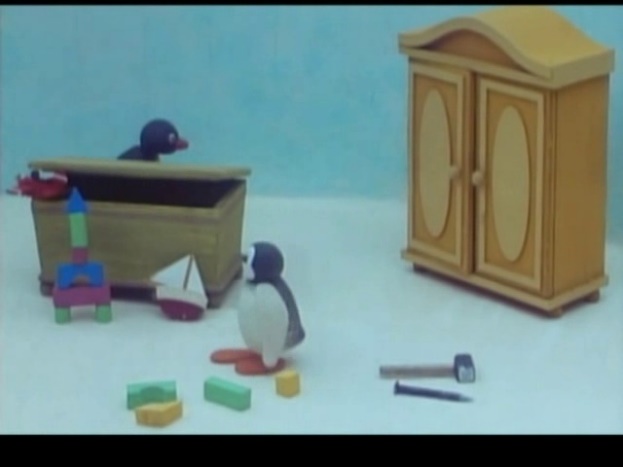 |

# *Table S2*. Comparison of model fits: pupillary components

| model | df | CFI | RMSEA | RMSEA CI-2.5% | RMSEA CI-97.5% |
| --- | --- | --- | --- | --- | --- |
| one component | 1175 | 0.950 | 0.061 | 0.058 | 0.064 |
| two components | 1174 | 0.954 | 0.059 | 0.055 | 0.062 |
| three components | 1172 | 0.958 | 0.056 | 0.053 | 0.059 |

| *Table S3*. Comparison of polynomial fits: pupillary response. | | | | | | | | |
| --- | --- | --- | --- | --- | --- | --- | --- | --- |
| model | parameters (n) | AIC | BIC | Log Lik | deviance | Chi-Square | df | p-value |
| first degree | 15 | 171301.1 | 171437.9 | -85635.57 | 171271.1 | NA | NA | NA |
| second degree | 16 | 171301.2 | 171447.0 | -85634.60 | 171269.2 | 1.940 | 1 | 0.164 |
| third degree | 17 | 171294.5 | 171449.5 | -85630.27 | 171260.5 | 8.657 | 1 | 0.003 |
| fourth degree | 18 | 171295.9 | 171460.0 | -85629.95 | 171259.9 | 0.643 | 1 | 0.423 |

| *Table S4*. Model definitions and syntax. | | | |
| --- | --- | --- | --- |
| Model | | Definition | Syntax (lmer package in R) |
| A.  sensory salience | 1 | **dependent variable:** physical salience | scale(lowlvl_salience)~ |
|  |  | **fixed effects:** group, pupillary response, video category, time | t1_diagnosis*(rpd_z)*vid_social* (scale(ts.scene)+scale(I(ts.scene^2))+scale(I(ts.scene^3)))+ |
|  |  | **covariates:** sex, age, perceptual IQ, ADHD inattention symptoms, anxiety symptoms, depressive symptoms, eye-tracking data accuracy, eye-tracking data precision, center deviation of gaze, luminance | t1_sex+scale(t1_ageyrs)+scale(t1_piq)+ scale(adhd_inatt)+scale(anx_beck)+scale(dep_beck)+ scale(Accuracy)+scale(Precision)+scale(centdev)+ scale(luminance)+d |
|  |  | **random effects:** participant, scene | (1\|id)+(1\|vid_scene) |
|  | 2 | **dependent variable:** motion salience | scale(motion_salience)~ |
|  |  | **fixed effects:** group, pupillary response, video category, time | t1_diagnosis*(rpd_z)*vid_social* (scale(ts.scene)+scale(I(ts.scene^2))+scale(I(ts.scene^3)))+ |
|  |  | **covariates:** sex, age, perceptual IQ, ADHD inattention symptoms, anxiety symptoms, depressive symptoms, eye-tracking data accuracy, eye-tracking data precision, center deviation of gaze, luminance | t1_sex+scale(t1_ageyrs)+scale(t1_piq)+ scale(adhd_inatt)+scale(anx_beck)+scale(dep_beck)+ scale(Accuracy)+scale(Precision)+scale(centdev)+ scale(luminance)+ |
|  |  | **random effects:** participant, scene | (1\|id)+(1\|vid_scene) |
| B. pupillary response | 3 | **dependent variable:** pupillary response | scale(rpd)~ |
|  |  | **fixed effects:** group, video category, time | t1_diagnosis*vid_social* (scale(ts.scene)+scale(I(ts.scene^2))+scale(I(ts.scene^3)))+ |
|  |  | **covariates:** sex, age, perceptual IQ, ADHD inattention symptoms, anxiety symptoms, depressive symptoms, eye-tracking data accuracy, eye-tracking data precision, center deviation of gaze, luminance | t1_sex+scale(t1_ageyrs)+scale(t1_piq)+ scale(adhd_inatt)+scale(anx_beck)+scale(dep_beck)+ scale(Accuracy)+scale(Precision)+scale(centdev)+ scale(luminance)+ |
|  |  | **random effects:** participant, scene | (1\|id)+(1\|vid_scene) |
| C.  social attention | 4 | **dependent variable:** social attention | scale(rpd)~ |
|  |  | **fixed effects:** group, time | t1_diagnosis* (scale(ts.scene)+scale(I(ts.scene^2))+scale(I(ts.scene^3)))+ |
|  |  | **covariates:** sex, age, perceptual IQ, ADHD inattention symptoms, anxiety symptoms, depressive symptoms, eye-tracking data accuracy, eye-tracking data precision, center deviation of gaze, luminance | t1_sex+scale(t1_ageyrs)+scale(t1_piq)+ scale(adhd_inatt)+scale(anx_beck)+scale(dep_beck)+ scale(Accuracy)+scale(Precision)+scale(centdev)+ scale(luminance)+ |
|  |  | **random effects:** participant, scene | (1\|id)+(1\|vid_scene) |
|  | 5 | **dependent variable:** social attention | scale(aoi_face)~ |
|  |  | **fixed effects:** group, motion salience, physical salience, time | t1_diagnosis*(motion_salience_z+lowlvl_salience_z)* (scale(ts.scene)+scale(I(ts.scene^2))+scale(I(ts.scene^3)))+ |
|  |  | **covariates:** sex, age, perceptual IQ, ADHD inattention symptoms, anxiety symptoms, depressive symptoms, eye-tracking data accuracy, eye-tracking data precision, center deviation of gaze, luminance | t1_sex+scale(t1_ageyrs)+scale(t1_piq)+ scale(adhd_inatt)+scale(anx_beck)+scale(dep_beck)+ scale(Accuracy)+scale(Precision)+scale(centdev)+ scale(luminance)+ |
|  |  | **random effects:** participant, scene | (1\|id)+(1\|vid_scene) |
|  | 6 | **dependent variable:** social attention | scale(aoi_face)~ |
|  |  | **fixed effects:** group, pupillary response, time | t1_diagnosis*(rpd_z)* (scale(ts.scene)+scale(I(ts.scene^2))+scale(I(ts.scene^3)))+ |
|  |  | **covariates:** sex, age, perceptual IQ, ADHD inattention symptoms, anxiety symptoms, depressive symptoms, eye-tracking data accuracy, eye-tracking data precision, center deviation of gaze, luminance | t1_sex+scale(t1_ageyrs)+scale(t1_piq)+ scale(adhd_inatt)+scale(anx_beck)+scale(dep_beck)+ scale(Accuracy)+scale(Precision)+scale(centdev)+scale(luminance)+ |
|  |  | **random effects:** participant, scene | (1\|id)+(1\|vid_scene) |

| *Table S5*. Linear mixed model: physical salience. | | | | | | |
| --- | --- | --- | --- | --- | --- | --- |
|  | Sum Sq | df1 | df2 | F | p | p_adj |
| group | 4.161 | 1 | 36900.774 | 4.821 | 0.028 | 0.084 |
| pupillary response (PR) | 18.289 | 1 | 51985.857 | 21.192 | 0.000 | 0.000 |
| video category (VC) | 13.090 | 1 | 301.724 | 15.168 | 0.000 | 0.000 |
| time^1 | 0.154 | 1 | 66241.651 | 0.178 | 0.673 | 1.000 |
| time^2 | 0.005 | 1 | 66262.224 | 0.006 | 0.941 | 1.000 |
| time^3 | 0.020 | 1 | 66271.758 | 0.024 | 0.878 | 1.000 |
| sex | 0.002 | 1 | 243.428 | 0.002 | 0.964 | 1.000 |
| age | 0.260 | 1 | 271.043 | 0.301 | 0.584 | 1.000 |
| perceptual IQ | 1.066 | 1 | 258.014 | 1.235 | 0.267 | 0.801 |
| ADHD inattention | 2.647 | 1 | 268.196 | 3.068 | 0.081 | 0.243 |
| anxiety | 0.311 | 1 | 210.339 | 0.360 | 0.549 | 1.000 |
| depression | 0.017 | 1 | 219.515 | 0.020 | 0.888 | 1.000 |
| accuracy | 1.510 | 1 | 326.366 | 1.749 | 0.187 | 0.561 |
| precision | 0.018 | 1 | 367.513 | 0.021 | 0.885 | 1.000 |
| center deviation | 729.942 | 1 | 57851.096 | 845.822 | 0.000 | 0.000 |
| luminance | 558.547 | 1 | 65720.243 | 647.218 | 0.000 | 0.000 |
| group x PR | 3.247 | 1 | 55354.862 | 3.763 | 0.052 | 0.156 |
| group x VC | 1.031 | 1 | 27921.298 | 1.195 | 0.274 | 0.822 |
| PR x VC | 7.537 | 1 | 27988.163 | 8.734 | 0.003 | 0.009 |
| group x time^1 | 0.573 | 1 | 66234.962 | 0.664 | 0.415 | 1.000 |
| group x time^2 | 0.110 | 1 | 66257.853 | 0.127 | 0.722 | 1.000 |
| group x time^3 | 0.001 | 1 | 66268.431 | 0.001 | 0.975 | 1.000 |
| PR x time^1 | 0.001 | 1 | 66244.556 | 0.001 | 0.977 | 1.000 |
| PR x time^2 | 0.062 | 1 | 66264.944 | 0.072 | 0.788 | 1.000 |
| PR x time^3 | 0.073 | 1 | 67455.560 | 0.085 | 0.771 | 1.000 |
| VC x time^1 | 0.678 | 1 | 67447.563 | 0.785 | 0.376 | 1.000 |
| VC x time^2 | 0.222 | 1 | 67460.945 | 0.257 | 0.612 | 1.000 |
| VC x time^3 | 0.252 | 1 | 67459.443 | 0.292 | 0.589 | 1.000 |
| group x PR x VC | 0.067 | 1 | 31005.862 | 0.078 | 0.780 | 1.000 |
| group x PR x time^1 | 0.088 | 1 | 67423.880 | 0.102 | 0.749 | 1.000 |
| group x PR x time^2 | 0.000 | 1 | 67446.835 | 0.000 | 0.994 | 1.000 |
| group x PR x time^3 | 0.014 | 1 | 66271.441 | 0.016 | 0.898 | 1.000 |
| group x VC x time^1 | 2.485 | 1 | 66258.918 | 2.879 | 0.090 | 0.270 |
| group x VC x time^2 | 2.275 | 1 | 66269.709 | 2.637 | 0.104 | 0.312 |
| group x VC x time^3 | 1.970 | 1 | 66267.838 | 2.283 | 0.131 | 0.393 |
| PR x VC x time^1 | 0.156 | 1 | 66272.001 | 0.181 | 0.671 | 1.000 |
| PR x VC x time^2 | 0.062 | 1 | 66281.108 | 0.071 | 0.789 | 1.000 |
| PR x VC x time^3 | 0.150 | 1 | 66275.762 | 0.174 | 0.677 | 1.000 |
| group x PR x VC x time^1 | 2.541 | 1 | 66258.969 | 2.944 | 0.086 | 0.258 |
| group x PR x VC x time^2 | 2.253 | 1 | 66269.670 | 2.610 | 0.106 | 0.318 |
| group x PR x VC x time^3 | 1.904 | 1 | 66266.817 | 2.207 | 0.137 | 0.411 |

*Note.* Group = Autism Spectrum Disorder, Neurotypical Development; video category = scenes with humans, scenes without humans; time^1 = linear effect of time, time^2 = quadratic effect of time, time^3 = cubic effect of time, ADHD inattention score = inattention subscale of the DSM-5 ADHD rating scale, anxiety = Beck`s anxiety inventory (BAI) total score, depression = Beck`s depression inventory (BDI) total score.

| *Table S6*. Linear mixed model: motion salience. | | | | | | |
| --- | --- | --- | --- | --- | --- | --- |
|  | Sum Sq | df1 | df2 | F | p | p_adj |
| group | 0.003 | 1 | 33463.494 | 0.003 | 0.954 | 1.000 |
| pupillary response (PR) | 1.022 | 1 | 40360.754 | 1.169 | 0.280 | 0.840 |
| video category (VC) | 4.607 | 1 | 280.191 | 5.269 | 0.022 | 0.066 |
| time^1 | 2.358 | 1 | 66313.932 | 2.697 | 0.101 | 0.303 |
| time^2 | 1.377 | 1 | 66325.339 | 1.575 | 0.209 | 0.627 |
| time^3 | 0.759 | 1 | 66328.112 | 0.868 | 0.352 | 1.000 |
| sex | 0.046 | 1 | 237.435 | 0.053 | 0.818 | 1.000 |
| age | 0.432 | 1 | 271.002 | 0.494 | 0.483 | 1.000 |
| perceptual IQ | 0.159 | 1 | 246.946 | 0.181 | 0.671 | 1.000 |
| ADHD inattention | 1.154 | 1 | 274.373 | 1.320 | 0.252 | 0.756 |
| anxiety | 0.002 | 1 | 194.158 | 0.002 | 0.965 | 1.000 |
| depression | 0.033 | 1 | 209.154 | 0.038 | 0.847 | 1.000 |
| accuracy | 1.814 | 1 | 385.650 | 2.075 | 0.151 | 0.453 |
| precision | 1.695 | 1 | 452.268 | 1.939 | 0.164 | 0.492 |
| center deviation | 108.924 | 1 | 46489.761 | 124.576 | 0.000 | 0.000 |
| luminance | 70.351 | 1 | 65579.309 | 80.460 | 0.000 | 0.000 |
| group x PR | 0.008 | 1 | 44603.794 | 0.009 | 0.925 | 1.000 |
| group x VC | 0.130 | 1 | 16186.913 | 0.149 | 0.700 | 1.000 |
| PR x VC | 2.927 | 1 | 14966.322 | 3.348 | 0.067 | 0.201 |
| group x time^1 | 0.434 | 1 | 66308.115 | 0.496 | 0.481 | 1.000 |
| group x time^2 | 0.109 | 1 | 66318.351 | 0.125 | 0.724 | 1.000 |
| group x time^3 | 0.014 | 1 | 66320.616 | 0.017 | 0.898 | 1.000 |
| PR x time^1 | 0.001 | 1 | 66315.949 | 0.002 | 0.967 | 1.000 |
| PR x time^2 | 0.228 | 1 | 66326.676 | 0.261 | 0.610 | 1.000 |
| PR x time^3 | 0.528 | 1 | 66328.742 | 0.604 | 0.437 | 1.000 |
| VC x time^1 | 34.140 | 1 | 66326.787 | 39.046 | 0.000 | 0.000 |
| VC x time^2 | 45.766 | 1 | 66330.000 | 52.343 | 0.000 | 0.000 |
| VC x time^3 | 50.257 | 1 | 66329.364 | 57.478 | 0.000 | 0.000 |
| group x PR x VC | 0.074 | 1 | 16928.528 | 0.084 | 0.772 | 1.000 |
| group x PR x time^1 | 0.433 | 1 | 66310.408 | 0.495 | 0.482 | 1.000 |
| group x PR x time^2 | 0.134 | 1 | 66319.513 | 0.153 | 0.695 | 1.000 |
| group x PR x time^3 | 0.030 | 1 | 66320.848 | 0.034 | 0.853 | 1.000 |
| group x VC x time^1 | 1.488 | 1 | 66318.039 | 1.701 | 0.192 | 0.576 |
| group x VC x time^2 | 2.921 | 1 | 66321.101 | 3.340 | 0.068 | 0.204 |
| group x VC x time^3 | 4.111 | 1 | 66320.988 | 4.702 | 0.030 | 0.090 |
| PR x VC x time^1 | 30.477 | 1 | 66327.539 | 34.856 | 0.000 | 0.000 |
| PR x VC x time^2 | 42.218 | 1 | 66330.627 | 48.284 | 0.000 | 0.000 |
| PR x VC x time^3 | 46.813 | 1 | 66329.541 | 53.540 | 0.000 | 0.000 |
| group x PR x VC x time^1 | 1.247 | 1 | 66318.223 | 1.426 | 0.232 | 0.696 |
| group x PR x VC x time^2 | 2.518 | 1 | 66321.195 | 2.880 | 0.090 | 0.270 |
| group x PR x VC x time^3 | 3.575 | 1 | 66321.013 | 4.089 | 0.043 | 0.129 |

*Note.* Group = Autism Spectrum Disorder, Neurotypical Development; video category = scenes with humans, scenes without humans; time^1 = linear effect of time, time^2 = quadratic effect of time, time^3 = cubic effect of time, ADHD inattention score = inattention subscale of the DSM-5 ADHD rating scale, anxiety = Beck`s anxiety inventory (BAI) total score, depression = Beck`s depression inventory (BDI) total score.

| *Table S7*. Linear mixed model: pupillary response. | | | | | | |
| --- | --- | --- | --- | --- | --- | --- |
|  | Sum Sq | df1 | df2 | F | p | p_adj |
| group | 0.352 | 1 | 306.320 | 0.503 | 0.479 | 1.000 |
| video category (VC) | 14.003 | 1 | 82.072 | 20.006 | 0.000 | 0.000 |
| time^1 | 2.276 | 1 | 66070.664 | 3.252 | 0.071 | 0.213 |
| time^2 | 2.661 | 1 | 66073.450 | 3.802 | 0.051 | 0.153 |
| time^3 | 2.092 | 1 | 66071.851 | 2.989 | 0.084 | 0.252 |
| sex | 0.037 | 1 | 308.642 | 0.053 | 0.818 | 1.000 |
| age | 0.526 | 1 | 315.997 | 0.752 | 0.387 | 1.000 |
| perceptual IQ | 2.137 | 1 | 312.918 | 3.054 | 0.082 | 0.246 |
| ADHD inattention | 0.001 | 1 | 313.389 | 0.002 | 0.967 | 1.000 |
| anxiety | 3.189 | 1 | 302.898 | 4.556 | 0.034 | 0.102 |
| depression | 1.635 | 1 | 303.728 | 2.336 | 0.127 | 0.381 |
| accuracy | 0.102 | 1 | 316.725 | 0.145 | 0.703 | 1.000 |
| precision | 0.029 | 1 | 330.433 | 0.041 | 0.839 | 1.000 |
| center deviation | 213.172 | 1 | 66333.815 | 304.544 | 0.000 | 0.000 |
| luminance | 4.068 | 1 | 66177.836 | 5.811 | 0.016 | 0.048 |
| group x VC | 135.058 | 1 | 66329.212 | 192.948 | 0.000 | 0.000 |
| group x time^1 | 1.450 | 1 | 66049.398 | 2.071 | 0.150 | 0.450 |
| group x time^2 | 1.328 | 1 | 66051.564 | 1.897 | 0.168 | 0.504 |
| group x time^3 | 0.798 | 1 | 66052.011 | 1.140 | 0.286 | 0.858 |
| VC x time^1 | 0.218 | 1 | 66068.790 | 0.311 | 0.577 | 1.000 |
| VC x time^2 | 0.263 | 1 | 66071.001 | 0.376 | 0.540 | 1.000 |
| VC x time^3 | 0.597 | 1 | 66070.934 | 0.853 | 0.356 | 1.000 |
| group x VC x time^1 | 5.877 | 1 | 66051.529 | 8.396 | 0.004 | 0.012 |
| group x VC x time^2 | 6.025 | 1 | 66055.550 | 8.608 | 0.003 | 0.009 |
| group x VC x time^3 | 4.501 | 1 | 66055.865 | 6.430 | 0.011 | 0.033 |

*Note.* Group = Autism Spectrum Disorder, Neurotypical Development; video category = scenes with humans, scenes without humans; time^1 = linear effect of time, time^2 = quadratic effect of time, time^3 = cubic effect of time, ADHD inattention score = inattention subscale of the DSM-5 ADHD rating scale, anxiety = Beck`s anxiety inventory (BAI) total score, depression = Beck`s depression inventory (BDI) total score.

| *Table S8*. Linear mixed model: social attention by group. | | | | | | |
| --- | --- | --- | --- | --- | --- | --- |
|  | Sum Sq | df1 | df2 | F | p | p_adj |
| group | 2.875 | 1 | 289.720 | 5.081 | 0.025 | 0.075 |
| time^1 | 509.048 | 1 | 30128.451 | 899.526 | 0.000 | 0.000 |
| time^2 | 407.051 | 1 | 30132.887 | 719.290 | 0.000 | 0.000 |
| time^3 | 347.632 | 1 | 30133.326 | 614.293 | 0.000 | 0.000 |
| sex | 2.638 | 1 | 290.430 | 4.662 | 0.032 | 0.096 |
| age | 14.954 | 1 | 294.300 | 26.424 | 0.000 | 0.000 |
| perceptual IQ | 5.523 | 1 | 292.819 | 9.760 | 0.002 | 0.006 |
| ADHD inattention | 1.586 | 1 | 292.534 | 2.803 | 0.095 | 0.285 |
| anxiety | 2.131 | 1 | 277.863 | 3.766 | 0.053 | 0.159 |
| depression | 2.916 | 1 | 281.823 | 5.152 | 0.024 | 0.072 |
| accuracy | 4.037 | 1 | 298.437 | 7.134 | 0.008 | 0.024 |
| precision | 1.161 | 1 | 318.969 | 2.052 | 0.153 | 0.459 |
| center deviation | 0.545 | 1 | 30375.752 | 0.964 | 0.326 | 0.978 |
| luminance | 1.925 | 1 | 30321.615 | 3.402 | 0.065 | 0.195 |
| group x time^1 | 0.014 | 1 | 30127.017 | 0.025 | 0.874 | 1.000 |
| group x time^2 | 0.003 | 1 | 30131.480 | 0.005 | 0.944 | 1.000 |
| group x time^3 | 0.005 | 1 | 30130.469 | 0.009 | 0.923 | 1.000 |

*Note.* Group = Autism Spectrum Disorder, Neurotypical Development; time^1 = linear effect of time, time^2 = quadratic effect of time, time^3 = cubic effect of time, ADHD inattention score = inattention subscale of the DSM-5 ADHD rating scale, anxiety = Beck`s anxiety inventory (BAI) total score, depression = Beck`s depression inventory (BDI) total score.

| *Table S9*. Linear mixed model: social attention by group without mediators and covariates. | | | | | | |
| --- | --- | --- | --- | --- | --- | --- |
|  | Sum Sq | df1 | df2 | F | p | p_adj |
| roup | 9.788 | 1 | 298.449 | 17.296 | 0.000 | 0.000 |
| time^1 | 508.779 | 1 | 30125.400 | 899.066 | 0.000 | 0.000 |
| time^2 | 406.789 | 1 | 30130.355 | 718.839 | 0.000 | 0.000 |
| time^3 | 347.492 | 1 | 30131.056 | 614.056 | 0.000 | 0.000 |
| group x time^1 | 0.012 | 1 | 30124.881 | 0.021 | 0.885 | 1.000 |
| group x time^2 | 0.002 | 1 | 30128.992 | 0.004 | 0.953 | 1.000 |
| group x time^3 | 0.004 | 1 | 30128.239 | 0.007 | 0.931 | 1.000 |

*Note.* Group = Autism Spectrum Disorder, Neurotypical Development; time^1 = linear effect of time, time^2 = quadratic effect of time, time^3 = cubic effect of time.

| *Table S10.* Linear mixed model: social attention with gazes on physical salience as mediator. | | | | | | |
| --- | --- | --- | --- | --- | --- | --- |
|  | Sum Sq | df1 | df2 | F | p | p_adj |
| group | 2.780 | 1 | 290.787 | 4.921 | 0.027 | 0.081 |
| physical salience (PS) | 8.634 | 1 | 30175.268 | 15.283 | 0.000 | 0.000 |
| time^1 | 459.000 | 1 | 30124.657 | 812.442 | 0.000 | 0.000 |
| time^2 | 365.226 | 1 | 30128.507 | 646.459 | 0.000 | 0.000 |
| time^3 | 311.647 | 1 | 30128.370 | 551.624 | 0.000 | 0.000 |
| sex | 2.629 | 1 | 290.339 | 4.653 | 0.032 | 0.096 |
| age | 14.857 | 1 | 294.203 | 26.298 | 0.000 | 0.000 |
| perceptual IQ | 5.552 | 1 | 292.754 | 9.828 | 0.002 | 0.006 |
| ADHD inattention | 1.548 | 1 | 292.440 | 2.739 | 0.099 | 0.297 |
| anxiety | 2.188 | 1 | 277.812 | 3.873 | 0.050 | 0.150 |
| depression | 2.955 | 1 | 281.747 | 5.230 | 0.023 | 0.069 |
| accuracy | 4.090 | 1 | 298.315 | 7.239 | 0.008 | 0.024 |
| precision | 1.220 | 1 | 318.858 | 2.159 | 0.143 | 0.429 |
| center deviation | 0.142 | 1 | 30364.490 | 0.252 | 0.616 | 1.000 |
| luminance | 3.141 | 1 | 30310.564 | 5.560 | 0.018 | 0.054 |
| group x PS | 0.001 | 1 | 30195.814 | 0.001 | 0.975 | 1.000 |
| group x time^1 | 0.000 | 1 | 30122.715 | 0.000 | 0.989 | 1.000 |
| group x time^2 | 0.004 | 1 | 30126.714 | 0.008 | 0.931 | 1.000 |
| group x time^3 | 0.001 | 1 | 30125.071 | 0.002 | 0.964 | 1.000 |
| PS x time^1 | 20.982 | 1 | 30134.409 | 37.139 | 0.000 | 0.000 |
| PS x time^2 | 23.289 | 1 | 30127.762 | 41.222 | 0.000 | 0.000 |
| PS x time^3 | 23.990 | 1 | 30123.780 | 42.463 | 0.000 | 0.000 |
| group x PS x time^1 | 0.000 | 1 | 30132.373 | 0.000 | 0.994 | 1.000 |
| group x PS x time^2 | 0.008 | 1 | 30126.361 | 0.014 | 0.907 | 1.000 |
| group x PS x time^3 | 0.021 | 1 | 30122.620 | 0.038 | 0.845 | 1.000 |

*Note.* Group = Autism Spectrum Disorder, Neurotypical Development; time^1 = linear effect of time, time^2 = quadratic effect of time, time^3 = cubic effect of time, ADHD inattention score = inattention subscale of the DSM-5 ADHD rating scale, anxiety = Beck`s anxiety inventory (BAI) total score, depression = Beck`s depression inventory (BDI) total score.

| *Table S11*. Linear mixed model: gazes on physical salience associated with group. | | | | | | |
| --- | --- | --- | --- | --- | --- | --- |
|  | Sum Sq | df1 | df2 | F | p | p_adj |
| group | 7.530 | 1 | 223.214 | 8.582 | 0.004 | 0.012 |
| time^1 | 5.663 | 1 | 30345.193 | 6.455 | 0.011 | 0.033 |
| time^2 | 5.863 | 1 | 30355.074 | 6.682 | 0.010 | 0.030 |
| time^3 | 4.996 | 1 | 30352.256 | 5.694 | 0.017 | 0.051 |
| sex | 0.056 | 1 | 226.075 | 0.064 | 0.801 | 1.000 |
| age | 0.168 | 1 | 249.363 | 0.191 | 0.662 | 1.000 |
| perceptual IQ | 5.811 | 1 | 233.764 | 6.622 | 0.011 | 0.033 |
| ADHD inattention | 0.333 | 1 | 250.633 | 0.379 | 0.539 | 1.000 |
| anxiety | 5.495 | 1 | 189.201 | 6.263 | 0.013 | 0.039 |
| depression | 0.902 | 1 | 203.264 | 1.028 | 0.312 | 0.936 |
| accuracy | 0.022 | 1 | 308.227 | 0.026 | 0.873 | 1.000 |
| precision | 0.475 | 1 | 341.253 | 0.541 | 0.463 | 1.000 |
| center deviation | 507.199 | 1 | 27406.570 | 578.052 | 0.000 | 0.000 |
| luminance | 538.366 | 1 | 30048.402 | 613.572 | 0.000 | 0.000 |
| group x time^1 | 1.466 | 1 | 30330.214 | 1.671 | 0.196 | 0.588 |
| group x time^2 | 1.273 | 1 | 30336.771 | 1.451 | 0.228 | 0.684 |
| group x time^3 | 1.075 | 1 | 30332.550 | 1.225 | 0.268 | 0.804 |

*Note.* Group = Autism Spectrum Disorder, Neurotypical Development; time^1 = linear effect of time, time^2 = quadratic effect of time, time^3 = cubic effect of time, ADHD inattention score = inattention subscale of the DSM-5 ADHD rating scale, anxiety = Beck`s anxiety inventory (BAI) total score, depression = Beck`s depression inventory (BDI) total score.

| *Table S12*. Linear mixed model: motion salience associated with group. | | | | | | |
| --- | --- | --- | --- | --- | --- | --- |
|  | Sum Sq | df1 | df2 | F | p | p_adj |
| group | 0.107 | 1 | 206.931 | 0.122 | 0.727 | 1.000 |
| time^1 | 97.596 | 1 | 31096.609 | 111.810 | 0.000 | 0.000 |
| time^2 | 98.733 | 1 | 31097.050 | 113.112 | 0.000 | 0.000 |
| time^3 | 94.011 | 1 | 31099.977 | 107.702 | 0.000 | 0.000 |
| sex | 0.050 | 1 | 214.233 | 0.057 | 0.811 | 1.000 |
| age | 0.630 | 1 | 235.231 | 0.722 | 0.396 | 1.000 |
| perceptual IQ | 0.048 | 1 | 219.784 | 0.055 | 0.814 | 1.000 |
| ADHD inattention | 0.293 | 1 | 243.446 | 0.336 | 0.563 | 1.000 |
| anxiety | 1.191 | 1 | 168.926 | 1.364 | 0.244 | 0.732 |
| depression | 2.957 | 1 | 190.995 | 3.387 | 0.067 | 0.201 |
| accuracy | 0.163 | 1 | 301.062 | 0.187 | 0.666 | 1.000 |
| precision | 0.064 | 1 | 379.471 | 0.073 | 0.787 | 1.000 |
| center deviation | 61.227 | 1 | 25748.607 | 70.144 | 0.000 | 0.000 |
| luminance | 26.304 | 1 | 120.590 | 30.135 | 0.000 | 0.000 |
| group x time^1 | 0.347 | 1 | 31044.889 | 0.398 | 0.528 | 1.000 |
| group x time^2 | 0.128 | 1 | 31053.081 | 0.147 | 0.701 | 1.000 |
| group x time^3 | 0.034 | 1 | 31052.912 | 0.039 | 0.843 | 1.000 |

*Note.* Group = Autism Spectrum Disorder, Neurotypical Development; time^1 = linear effect of time, time^2 = quadratic effect of time, time^3 = cubic effect of time, ADHD inattention score = inattention subscale of the DSM-5 ADHD rating scale, anxiety = Beck`s anxiety inventory (BAI) total score, depression = Beck`s depression inventory (BDI) total score.

| *Table S13*. Linear mixed model: social attention by group with pupillary response as moderator. | | | | | | |
| --- | --- | --- | --- | --- | --- | --- |
|  | Sum Sq | df1 | df2 | F | p | p_adj |
| group | 1.300 | 1 | 12006.391 | 2.296 | 0.130 | 0.390 |
| pupillary response (PR) | 3.561 | 1 | 28786.008 | 6.291 | 0.012 | 0.036 |
| time^1 | 23.131 | 1 | 30085.428 | 40.867 | 0.000 | 0.000 |
| time^2 | 18.844 | 1 | 30089.683 | 33.292 | 0.000 | 0.000 |
| time^3 | 15.716 | 1 | 30092.736 | 27.767 | 0.000 | 0.000 |
| sex | 2.714 | 1 | 290.906 | 4.796 | 0.029 | 0.087 |
| age | 14.855 | 1 | 294.692 | 26.245 | 0.000 | 0.000 |
| perceptual IQ | 5.704 | 1 | 293.463 | 10.077 | 0.002 | 0.006 |
| ADHD inattention | 1.667 | 1 | 292.939 | 2.946 | 0.087 | 0.261 |
| anxiety | 2.112 | 1 | 278.342 | 3.732 | 0.054 | 0.162 |
| depression | 2.842 | 1 | 282.227 | 5.021 | 0.026 | 0.078 |
| accuracy | 4.053 | 1 | 298.768 | 7.162 | 0.008 | 0.024 |
| precision | 1.246 | 1 | 319.344 | 2.202 | 0.139 | 0.417 |
| center deviation | 0.403 | 1 | 30327.296 | 0.713 | 0.399 | 1.000 |
| luminance | 2.038 | 1 | 30271.651 | 3.601 | 0.058 | 0.174 |
| group x PR | 3.172 | 1 | 29210.583 | 5.603 | 0.018 | 0.054 |
| group x time^1 | 0.516 | 1 | 30084.450 | 0.911 | 0.340 | 1.000 |
| group x time^2 | 0.535 | 1 | 30088.353 | 0.946 | 0.331 | 0.993 |
| group x time^3 | 0.532 | 1 | 30091.387 | 0.940 | 0.332 | 0.996 |
| PR x time^1 | 6.640 | 1 | 30085.689 | 11.731 | 0.001 | 0.003 |
| PR x time^2 | 5.437 | 1 | 30088.913 | 9.605 | 0.002 | 0.006 |
| PR x time^3 | 4.432 | 1 | 30091.173 | 7.831 | 0.005 | 0.015 |
| group x PR x time^1 | 0.504 | 1 | 30084.501 | 0.891 | 0.345 | 1.000 |
| group x PR x time^2 | 0.516 | 1 | 30087.468 | 0.912 | 0.340 | 1.000 |
| group x PR x time^3 | 0.518 | 1 | 30089.691 | 0.915 | 0.339 | 1.000 |

*Note.* Group = Autism Spectrum Disorder, Neurotypical Development; time^1 = linear effect of time, time^2 = quadratic effect of time, time^3 = cubic effect of time, ADHD inattention score = inattention subscale of the DSM-5 ADHD rating scale, anxiety = Beck`s anxiety inventory (BAI) total score, depression = Beck`s depression inventory (BDI) total score.

| *Table S14*. Linear mixed model: social attention by group with pupillary components as moderators. | | | | | | |
| --- | --- | --- | --- | --- | --- | --- |
|  | Sum Sq | df1 | df2 | F | p | p_adj |
| group | 2.354 | 1 | 295.668 | 4.028 | 0.046 | 0.138 |
| early pupillary comp. (PC1) | 22.746 | 1 | 30189.330 | 38.921 | 0.000 | 0.000 |
| late pupillary comp. (PC2) | 9.612 | 1 | 30261.472 | 16.447 | 0.000 | 0.000 |
| sex | 2.739 | 1 | 289.677 | 4.686 | 0.031 | 0.093 |
| age | 15.008 | 1 | 293.670 | 25.680 | 0.000 | 0.000 |
| perceptual IQ | 5.711 | 1 | 292.287 | 9.772 | 0.002 | 0.006 |
| ADHD inattention | 1.838 | 1 | 291.896 | 3.146 | 0.077 | 0.231 |
| anxiety | 1.997 | 1 | 276.950 | 3.417 | 0.066 | 0.198 |
| depression | 2.887 | 1 | 280.962 | 4.940 | 0.027 | 0.081 |
| accuracy | 3.880 | 1 | 297.863 | 6.639 | 0.010 | 0.030 |
| precision | 1.244 | 1 | 318.623 | 2.128 | 0.146 | 0.438 |
| center deviation | 0.608 | 1 | 30383.387 | 1.040 | 0.308 | 0.924 |
| luminance | 2.324 | 1 | 30329.019 | 3.976 | 0.046 | 0.138 |
| group x PC1 | 0.570 | 1 | 30270.383 | 0.976 | 0.323 | 0.969 |
| group x PC2 | 2.245 | 1 | 30275.335 | 3.841 | 0.050 | 0.150 |

*Note.* Group = Autism Spectrum Disorder, Neurotypical Development; ADHD inattention score = inattention subscale of the DSM-5 ADHD rating scale, anxiety = Beck`s anxiety inventory (BAI) total score, depression = Beck`s depression inventory (BDI) total score.

# *Supplemental Information S15.* Areas-of-interest (AOI) definition criteria.

We applied the software Apple Motion for drawing and to define key frames; this allows the interpolation of AOI for subsequent video frames. The body, face and hands AOIs are drawn so as to encompass that area. The facial features (eyes, nose, mouth) encompass those features, but extend outward to include emotionally expressive regions that border the features themselves. For example, the eye region includes eyebrows.


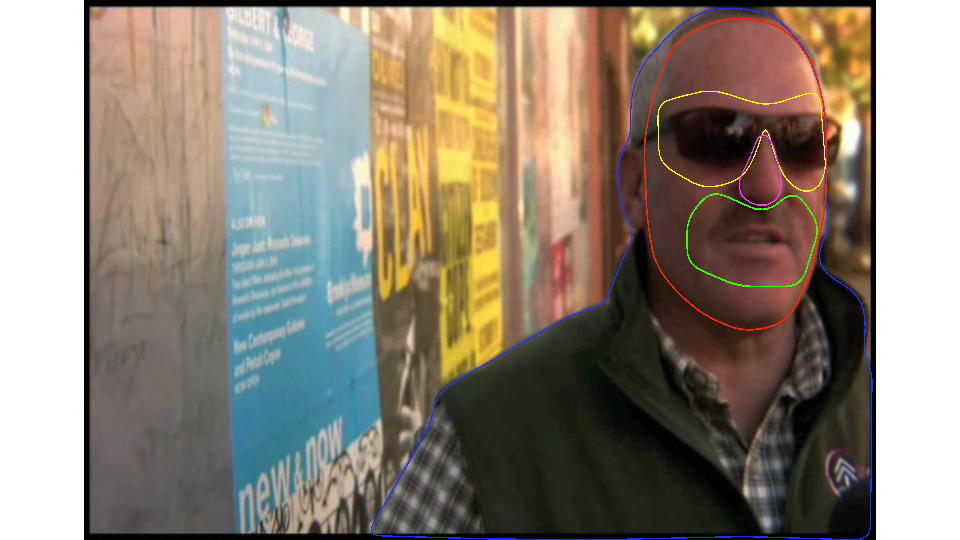


*Accuracy*

Aim to be as accurate as possible. Very small deviations (less than one degree of visual angle) on complex stimuli are acceptable as these are within the eye tracker’s spatial margin of error. If in doubt, be accurate.

*Face*

Follow the outline of the face, starting just below the hairline and following along the jawline.

*Body*

The entire silhouette of the individual. Including the arms, legs, torso, face and hair. You do not need to include hands, as these are a separate AOI.

*Eyes*

Encompasses the eyes and eyebrows, and includes the region of the forehead just above the eyes and the area to either side. The aim is to include emotionally expressive areas of the face related to the eyes.

*Mouth*

Include the mouth, upper and lower lips, and the emotionally expressive area above, below and to either side of the eyes. Note that the mouth AOI will move and change shape when the individual talks and their jaw extends downwards.

# *Supplemental Information S16.* Citations of applied R packages

1. Auguie B. gridExtra: Miscellaneous Functions for "Grid" Graphics; 2017. Available from: URL: https://CRAN.R-project.org/package=gridExtra.

2. Bartoń K. MuMIn: Multi-Model Inference; 2022. Available from: URL: https://CRAN.R-project.org/package=MuMIn.

3. Bates D, Mächler M, Bolker B, Walker S. Fitting Linear Mixed-Effects Models Using lme4. Journal of Statistical Software 2015; 67(1):1–48.

4. Bates D, Maechler M, Bolker B, Walker S. lme4: Linear Mixed-Effects Models using Eigen and S4; 2022. Available from: URL: https://github.com/lme4/lme4/.

5. Bates D, Maechler M, Jagan M. Matrix: Sparse and Dense Matrix Classes and Methods; 2022. Available from: URL: https://CRAN.R-project.org/package=Matrix.

6. Bengtsson H. R.matlab: Read and Write MAT Files and Call MATLAB from Within R; 2018. Available from: URL: https://github.com/HenrikBengtsson/R.matlab.

7. Blischak JD, Carbonetto P, Stephens M. Creating and sharing reproducible research code the workflowr way [version 1; peer review: 3 approved]. F1000Research 2019; 8(1749).

8. Blischak J, Carbonetto P, Stephens M. workflowr: A Framework for Reproducible and Collaborative Data Science; 2021. Available from: URL: https://github.com/workflowr/workflowr.

9. Carr D, Lewin-Koh N, Maechler M. hexbin: Hexagonal Binning Routines; 2021. Available from: URL: https://github.com/edzer/hexbin.

10. Chamberlain S, Zhu H, Jahn N, Boettiger C, Ram K. rcrossref: Client for Various CrossRef ’APIs’. Available from: URL: https://CRAN.R-project.org/package=rcrossref.

11. Eddelbuettel D. Seamless R and C++ Integration with Rcpp. New York: Springer; 2013.

12. Eddelbuettel D, Balamuta JJ. Extending extitR with extitC++: A Brief Introduction to extitRcpp. The American Statistician 2018; 72(1):28–36.

13. Eddelbuettel D, François R. Rcpp: Seamless R and C++ Integration. Journal of Statistical Software 2011; 40(8):1–18.

14. Eddelbuettel D, Francois R, Allaire JJ, Ushey K, Kou Q, Russell N et al. Rcpp: Seamless R and C++ Integration; 2022. Available from: URL: https://CRAN.R-project.org/package=Rcpp.

15. Gilleland E. Two-dimensional kernel smoothing: Using the R package smoothie. NCAR Technical Note, TN-502+STR, 17pp; 2013.

16. Gilleland E. smoothie: Two-Dimensional Field Smoothing; 2021. Available from: URL: https://ral.ucar.edu/staff/ericg/.

17. Gordon M. Gmisc: Descriptive Statistics, Transition Plots, and More; 2022. Available from: URL: https://gforge.se.

18. Gordon M, Gragg S, Konings P. htmlTable: Advanced Tables for Markdown/HTML; 2022. Available from: URL: https://gforge.se/packages/.

19. Green P, MacLeod CJ. simr: an R package for power analysis of generalised linear mixed models by simulation. Methods in Ecology and Evolution 2016; 7(4):493–8. Available from: URL: https://CRAN.R-project.org/package=simr.

20. Green P, MacLeod C. simr: Power Analysis for Generalised Linear Mixed Models by Simulation; 2022. Available from: URL: https://github.com/pitakakariki/simr.

21. Ho DE, Imai K, King G, Stuart EA. MatchIt: Nonparametric Preprocessing for Parametric Causal Inference. Journal of Statistical Software 2011; 42(8):1–28.

22. Ho D, Imai K, King G, Stuart E, Greifer N. MatchIt: Nonparametric Preprocessing for Parametric Causal Inference; 2022. Available from: URL: https://CRAN.R-project.org/package=MatchIt.

23. Husson F, Josse J. missMDA: Handling Missing Values with Multivariate Data Analysis; 2020. Available from: URL: http://factominer.free.fr/missMDA/index.html.

24. Jefferis G. readbitmap: Simple Unified Interface to Read Bitmap Images (BMP,JPEG,PNG,TIFF); 2018. Available from: URL: https://github.com/jefferis/readbitmap.

25. Josse J, Husson F. missMDA: A Package for Handling Missing Values in Multivariate Data Analysis. Journal of Statistical Software 2016; 70(1):1–31.

26. Kuznetsova A, Brockhoff PB, Christensen RHB. lmerTest Package: Tests in Linear Mixed Effects Models. Journal of Statistical Software 2017; 82(13):1–26.

27. Kuznetsova A, Brockhoff PB, Christensen RHB. lmerTest: Tests in Linear Mixed Effects Models; 2020. Available from: URL: https://github.com/runehaubo/lmerTestR.

28. Lenth RV. emmeans: Estimated Marginal Means, aka Least-Squares Means; 2022. Available from: URL: https://github.com/rvlenth/emmeans.

29. Lüdecke D. sjmisc: Data and Variable Transformation Functions. Journal of Open Source Software 2018; 3(26):754.

30. Lüdecke D. sjmisc: Data and Variable Transformation Functions; 2021. Available from: URL: https://strengejacke.github.io/sjmisc/.

31. Lüdecke D. sjPlot: Data Visualization for Statistics in Social Science; 2021. Available from: URL: https://strengejacke.github.io/sjPlot/.

32. Neuwirth E. RColorBrewer: ColorBrewer Palettes; 2022. Available from: URL: https://CRAN.R-project.org/package=RColorBrewer.

33. Revelle W. psych: Procedures for Psychological, Psychometric, and Personality Research; 2022. Available from: URL: https://personality-project.org/r/psych/ https://personality-project.org/r/psych-manual.pdf.

34. Rosseel Y. lavaan: An R Package for Structural Equation Modeling. Journal of Statistical Software 2012; 48(2):1–36.

35. Rosseel Y, Jorgensen TD, Rockwood N. lavaan: Latent Variable Analysis; 2022. Available from: URL: https://lavaan.ugent.be.

36. van Buuren S, Groothuis-Oudshoorn K. mice: Multivariate Imputation by Chained Equations in R. Journal of Statistical Software 2011; 45(3):1–67.

37. van Buuren S, Groothuis-Oudshoorn K. mice: Multivariate Imputation by Chained Equations; 2021. Available from: URL: https://CRAN.R-project.org/package=mice.

38. Warnes GR, Bolker B, Lumley T, team C. gtools: Various R Programming Tools; 2022. Available from: URL: https://github.com/r-gregmisc/gtools.

39. Wickham H. Reshaping Data with the reshape Package. Journal of Statistical Software 2007; 21(12):1–20. Available from: URL: http://www.jstatsoft.org/v21/i12/.

40. Wickham H. ggplot2: Elegant Graphics for Data Analysis. Springer-Verlag New York; 2016. Available from: URL: https://ggplot2.tidyverse.org.

41. Wickham H. reshape2: Flexibly Reshape Data: A Reboot of the Reshape Package; 2020. Available from: URL: https://github.com/hadley/reshape.

42. Wickham H, Bryan J. readxl: Read Excel Files; 2022. Available from: URL: https://CRAN.R-project.org/package=readxl.

43. Wickham H, Chang W, Henry L, Pedersen TL, Takahashi K, Wilke C et al. ggplot2: Create Elegant Data Visualisations Using the Grammar of Graphics; 2022. Available from: URL: https://CRAN.R-project.org/package=ggplot2.

44. Wickham H, Hester J, Bryan J. readr: Read Rectangular Text Data; 2022. Available from: URL: https://CRAN.R-project.org/package=readr.

45. Zeileis A, Grothendieck G. zoo: S3 Infrastructure for Regular and Irregular Time Series. Journal of Statistical Software 2005; 14(6):1–27.

46. Zeileis A, Grothendieck G, Ryan JA. zoo: S3 Infrastructure for Regular and Irregular Time Series (Z’s Ordered Observations); 2022. Available from: URL: https://zoo.R-Forge.R-project.org/.

47. Zhu H. kableExtra: Construct Complex Table with kable and Pipe Syntax; 2021. Available from: URL: https://CRAN.R-project.org/package=kableExtra.

# *Supplemental Information S17.* Description of covariates in the statistical analyses.

We controlled for demographics by fixed effect covariates of sex, age, and perceptual IQ. A previous analysis on oculomotor function in an overlapping sample did not find group differences between ASD and TD specific to age groups (i.e., children, adolescents, adults) . Thus, for reasons of parsimony, we collapsed the age groups and included age, in years, as a numeric covariate.

We further controlled for comorbid psychopathology by fixed effect covariates of ADHD inattention symptoms (ADHD rating scale inattention subscale), anxiety symptoms (Beck Anxiety Inventory total score) and depressive symptoms (Beck Depression Inventory total score). This was applied to control that observed group differences are not confounded by comorbidity. The screening measures of comorbid symptoms were optional in the assessment protocol and, thus, were missing in a proportion of the sample (DSM-5 ADHD rating scale = 17.1 %, Beck Anxiety Inventory = 23.1%, Beck Depression Inventory = 21.0%). We imputed these missing measures by multiple imputations of chained equations (MICE) with predictive mean matching based on all other demographic variables.

Data quality was controlled for by fixed effect covariates of eye-tracking data accuracy and eye-tracking data precision. In a post-hoc calibration procedure with up to six gaze-contingent target points, accuracy was defined as the Euclidean distance of gaze estimates and target location, whereas precision was defined as the Euclidean distance of gaze estimate coordinates per target location.

Gaze behavior was controlled for by a fixed effect covariate of center deviation of gaze. This covariate also considers central biases in visual attention (1) that characterized previous group differences in gaze behavior between ASD and TD during a previous analysis on SSP (2).

Luminance was controlled for by a fixed effect covariate of local luminance of the current gaze location. The mean global luminance progression differed between the naturalistic social and non-social video scenes (see Figure 4). Luminance characteristics induce pupillary light adaptation and thus need to be considered in the pupillary responses to sensory salience.

*References*

1. Nuthmann A, Einhäuser W, Schütz I. How Well Can Saliency Models Predict Fixation Selection in Scenes Beyond Central Bias? A New Approach to Model Evaluation Using Generalized Linear Mixed Models. Frontiers in Human Neuroscience. 2017;11(491).

2. Wang S, Jiang M, Duchesne Xavier M, Laugeson Elizabeth A, Kennedy Daniel P, Adolphs R, et al. Atypical Visual Saliency in Autism Spectrum Disorder Quantified through Model-Based Eye Tracking. Neuron. 2015;88(3):604-16.
